# Comparative Performance of Ionic and Agro-Physiological Traits for Detecting Salt Tolerance in Wheat Genotypes Grown in Real Field Conditions

**DOI:** 10.3390/life14111487

**Published:** 2024-11-15

**Authors:** Muhammad Usman Tahir, Salah El-Hendawy, Nasser Al-Suhaibani

**Affiliations:** Department of Plant Production, College of Food and Agriculture Sciences, King Saud University, KSA, P.O. Box 2460, Riyadh 11451, Saudi Arabia; mtahir@ksu.edu.sa (M.U.T.); nsuhaib@ksu.edu.sa (N.A.-S.)

**Keywords:** canopy temperature, chlorophyll content, heatmap clustering, maximal PSII photochemical efficiency, Na^+^ content, relative water content

## Abstract

Studying the physiological mechanisms underlying the traits associated with salt tolerance in genotypes could lead to the discovery of new genetic resources for salt tolerance. In this study, the mechanisms of salt tolerance were evaluated, based on ionic, physiological, and agronomic traits in four varieties that differ in their salt tolerance and in 18 F_8_ recombinant inbred lines (RILs) grown in real field conditions. The salt tolerance of plant materials was assessed under both normal (3.5 mM NaCl) and high salinity stress (150 mM NaCl) conditions for two consecutive years. Different growth and physiological traits were assessed 75 days after sowing, while ion contents in the shoots, grain yield, and its components were determined at the maturity stage. Multivariate analysis was used to conduct a comprehensive evaluation of salt tolerance across various genotypes and traits. The ANOVA results showed significant differences (*p* ≤ 0.05 and 0.001) among salinity, genotypes, and their interactions for all ionic and agro-physiological traits, with a few exceptions. Salinity stress resulted in a considerable increase in Na^+^ content and canopy temperature (CT), with a simultaneous decrease of 11.3% to 94.5% in other ionic and agro-physiological traits compared to the control treatment. However, the salt-tolerant genotypes showed minimal increases in Na^+^ content and CT, as well as decreases in other ionic and agro-physiological traits when compared to salt-sensitive genotypes under salinity stress. All ionic and agro-physiological traits exhibited strong correlations with each other under salinity stress, but these correlations were weak or insignificant under control conditions. The principal component analysis identified Na^+^ and CT as negative indicators and other ionic and agro-physiological traits as positive indicators for salt tolerance under salinity stress. The negative indicators were strongly linked to salt-sensitive genotypes, while the positive indicators were closely associated with salt-tolerant genotypes. Heatmap clustering, using multiple traits, successfully differentiated the salt-tolerant genotypes from the salt-sensitive ones. The salt-tolerant group showed a significant reduction in Na^+^ content by 36.9%, in CT by 10.0%, and in HI by 16.7%, along with an increase of 6.3–51.4% in other ionic and agro-physiological traits compared to the salt-sensitive group. In conclusion, the mechanisms associated with Na^+^ exclusion and high K^+^/Na^+^ and Ca^2+^/Na^+^ ratios, as well as chlorophyll and relative water content, along with low CT, resulted in significant improvements in growth and yield under salinity stress conditions. Given that the effectiveness of various ionic and agro-physiological traits in evaluating salt tolerance in wheat has been proven in real field conditions, these traits will play a key role in the development of salt-tolerant wheat genotypes.

## 1. Introduction

By 2050, it is anticipated that almost half of the world’s arable land will be impacted by salinity, posing a threat to global food security for an estimated population of 9.7 billion [1]. Most importantly, recent scientific evidence shows that approximately 12 million hectares of agricultural land are destroyed annually due to salinization, leading to a global economic loss of around USD 27.3 billion per year [2]. Salinity is becoming a significant issue in arid and semiarid regions where rainfall is significantly lower than evaporation rates. Additionally, the scarcity of water in these regions necessitates the use of saline groundwater for irrigation, resulting in a rapid increase in soil salinity levels. Even irrigation with water having an electrical conductivity as low as 0.5 dS m^−1^ can lead to salinity stress, as salt can accumulate in the topsoil after water evaporation [3]. In spite of the challenges presented by salinity, it is crucial to cultivate crops on salt-affected soil or use brackish water for irrigation to ensure food security, even if it results in reduced yields. Bread wheat (*Triticum aestivum* L.) is a vital cereal crop that is essential for feeding a large part of the world’s population. To meet rising demand, wheat production needs to double by 2050 [4]. However, the moderate tolerance of wheat to salinity stress presents a challenge, as high salinity levels can reduce wheat yield by over 60% [5,6]. Interestingly, research indicates that developing stress-tolerant wheat genotypes (both biotic and abiotic) can increase grain yield by up to 25% [7]. Therefore, enhancing the salt tolerance of wheat genotypes is a practical strategy to counter the adverse effects of salinity stress on final crop yield. Providing farmers with salt-tolerant wheat genotypes is a cost-effective, easily implemented, and sustainable solution in the long term, as opposed to expensive methods such as leaching or excessive gypsum application.

Identifying wheat genotypes with broad salt tolerance is essential for developing salt-tolerant varieties. Unfortunately, only a limited number of genotypes with this trait have been incorporated into breeding programs. Therefore, there is an urgent need to identify new germplasms with salt tolerance to increase genetic diversity and access elite resources with diverse genetic backgrounds [8,9]. However, assessing the salt tolerance of genotypes is challenging due to several factors, including: (1) the majority of salinity studies often being conducted in controlled environments like greenhouses and by hydroponics rather than in real and complex field conditions; (2) the limited use of physiological indicators for assessing salt tolerance [10,11,12]; (3) the frequent use of grain yield as a screening criterion for salt tolerance, in spite of its low heritability [9,11,13]; (4) an inadequate understanding of the complex mechanisms involved in salt tolerance; (5) a focus on the early growth stages (germination and seedling stages) for evaluating salt tolerance, overlooking variations across different growth stages [14]; (6) the infrequent use of multiple parameters and appropriate statistical tools for the accurate and efficient evaluation of salt tolerance [15,16,17,18]; and (7) the limited use of high-throughput phenotyping tools for assessing the salt tolerance of genotypes in a rapid and cost-effective manner [19].

High external salt concentration in the root zone leads to an excessive accumulation of Na^+^ in the shoots. This high Na^+^ absorption competes with the uptake of K^+^ and Ca^2+^, causing an imbalance in the ratio of these ions. To maintain a high intercellular K^+^/Na^+^ and Ca^2+^/Na^+^ ratio, it is important to prevent the influx of Na^+^ into the shoots and enhance the uptake of K^+^ and Ca^2+^. These physiological mechanisms are essential for conferring salinity stress tolerance in various crops. Therefore, the concentrations of Na^+^, K^+^, and Ca^2+^ and their ratios are important screening criteria for assessing salt tolerance in genotypes. Studies have shown a strong association between salt tolerance in plant genotypes and the concentrations of Na^+^, as well as the ratios of Na^+^/K^+^ and Na^+^/Ca^2+^ in different field crops [2,8,11,17,20]. In wheat, the majority of Na^+^ exclusion occurs by limiting Na^+^ uptake at the soil–root interface and xylem loading in the roots; alternatively, the application of some exogenous substances (phytohormones) can help in balancing ion content under salinity stress conditions [21,22]. Genotypes with lower Na^+^ concentrations show improved dry matter production, reduced leaf injuries, and a higher ratio of living to dead leaves, indicating better growth. This growth enhancement is likely attributed to a more balanced carbon level in genotypes with lower Na^+^ concentrations. A study on the families resulting from a cross between high- and low-Na^+^ genotypes revealed a strong correlation between shoot dry matter and leaf Na^+^ concentration [23]. This emphasizes the significance of Na^+^ concentration and its ratio with K^+^ and Ca^2+^ as useful screening criteria for the identification of genetic donors for salt tolerance in wheat.

Salt stress can hinder plant growth and productivity by reducing photosynthesis capacity. The strong correlation between growth and photosynthesis capacity under salinity stress emphasizes the importance of this trait in assessing the salt tolerance of genotypes. Salinity stress reduces photosynthesis by lowering the maximum quantum PSII photochemical efficiency (F_v_/F_m_) and by damaging photosynthetic pigments through Na^+^ accumulation, stomatal closure, and decreased leaf area. In this context, chlorophyll content and F_v_/F_m_ have been confirmed as the key physiological criteria for evaluating the salt tolerance of genotypes in several field crops [8,24,25,26]. Most importantly, the early detection of both traits is not only useful as a screening criterion for evaluating the salt tolerance of genotypes but can also help prevent the loss of plant biomass under high-salinity conditions [27]. Additionally, measuring F_v_/F_m_ provides a rapid, cost-effective, and non-destructive method to evaluate the salt tolerance of a large number of genotypes. Excessive salt in the root zone can cause osmotic stress in plants, making it difficult for them to absorb water from the soil [28]. This stress is similar to the effects of drought, leading to stomatal closure, reduced transpiration, lower plant water content, and elevated canopy foliage temperature. Orzechowska et al. [29] found a significant rise in canopy foliage temperature when plants were exposed to salinity stress for a short period. Similarly, Sirault et al. [30] demonstrated that saline-stressed plants exhibited stomatal closure and reduced transpiration rates, leading to an increase in canopy foliage temperature. In barley, stomatal conductance and leaf temperature are closely linked, with variations being influenced by genotype [30]. The strong correlation between salinity stress and traits like stomatal conductance, leaf water content, and canopy temperature suggests that these traits may be valuable screening criteria for assessing salt tolerance in genotypes and identifying potential genetic donors for salt tolerance.

Agronomic parameters such as plant biomass, leaf area, yield components, and harvest index can also be useful as selection criteria for evaluating salt tolerance and differentiating between genotypes under salinity stress. Plant biomass is a key indicator of economic viability in saline conditions. Research shows that high salt levels reduce wheat plant biomass, whereas salt-tolerant genotypes maintain higher plant biomass by sustaining photosynthesis, increasing carbohydrate supply to leaves, enhancing water uptake, and preventing Na^+^ buildup in leaves under salinity conditions [2,31,32,33]. One immediate reaction to salt stress is the restricted growth of leaf surfaces. Various studies have documented this phenomenon in field crops that have been exposed to salinity stress [8,10,20,34]. The osmotic and ionic components of salinity stress are likely the primary factors contributing to the reduction in leaf expansion, impacting cell turgor, division, and expansion [32,35,36,37]. Several studies have demonstrated that grain number (GN) is more important for final grain yield than thousand-grain weight (TGW) in genotypes exposed to drought or salinity stress [38,39]. Stress conditions can result in inadequate pollination, higher kernel abortion rates, and, ultimately, lower GN and yield [39]. Considering all the information mentioned above, several agronomic parameters could serve as useful screening criteria for the selection and improvement of salt-tolerant genotypes.

Breeders aim to combine multiple desirable traits in a new genotype to achieve high performance; selection based on only a few traits may not guarantee genetic gains in other important traits. Therefore, when evaluating salt tolerance in genotypes, it is important to apply appropriate statistical tools that can analyze multiple parameters simultaneously. This method aids in categorizing tested genotypes according to their salt tolerance levels. Multivariate analysis, including principal component analysis and heatmap cluster analysis, is a valuable statistical tool for evaluating crop stress performance. It helps identify key plant traits that improve stress tolerance and distinguish stress-tolerant genotypes based on various traits, salinity levels, and growth stages. This analysis also helps in understanding the different adaptation mechanisms regarding stress conditions [14,40,41]. Therefore, the objectives of this study were to (1) investigate the effects of salinity stress on the ionic, physiological, and agronomic traits of various wheat genotypes grown in real-field conditions and irrigated with saline water; (2) identify those ionic and agro-physiological traits that can serve as effective and reliable screening criteria for assessing the salt tolerance of genotypes in real field conditions; and (3) comprehensively assess the salt tolerance of genotypes in real field conditions by analyzing multiple traits using multivariate analysis.

## 2. Materials and Methods

### 2.1. Plant Materials and Experimental Conditions

This study involved 22 spring wheat genotypes, including 18 F_8_ recombinant inbred lines (RIL), their three parents (Sakha 93, Sids 1, and Sakha 61), and the commercial genotype Kawz. The salt tolerance levels of the three parents were previously assessed under controlled and field conditions, using various agronomic and physiological traits at different growth stages. Sakha 93, Sids 1, and Sakha 61 were classified as salt-tolerant, moderately salt-tolerant, and salt-sensitive genotypes, respectively [42,43]. Of these, 7 out of 18 RILs were from a cross of Sakha 93 and Sakha 61 (RIL1-group), and the other 11 were from a cross of Sakha 93 and Sids 1 (RIL2-group).

The salt tolerance of different wheat genotypes was assessed in real-field conditions at the experimental station of the Plant Production Department, College of Food and Agriculture Sciences, King Saud University, located in the middle of Saudi Arabia. The genotypes were evaluated during two consecutive winter seasons (2019–2020 and 2020–2021). The experimental farm has arid conditions with mean temperatures ranging from 12.9 °C to 32.2 °C during wheat growing seasons. Rainfall ranged from 4.0 mm to 20.0 mm, and relative humidity ranged from 20% to 50%. The soil texture is of sandy loam with an electrical conductivity (EC) of 1.12 dS m^−1^, pH of 7.85, a bulk density of 1.48 g cm^−3^, an organic matter content of 0.46%, and a field capacity of 0.101 m^3^ m^−3^.

### 2.2. Experimental Design, Salinity Treatments, and Agronomic Practices

The experimental design included randomized blocks in a split-plot scheme with three replicates. The main plots had two salinity treatments: control (≈0.35 dS m^−1^) and high salinity level (≈15.0 dS m^−1^). The subplots consisted of 22 tested wheat genotypes. All genotypes were initially irrigated with normal water for three weeks after sowing, to aid germination and seedling establishment. Subsequently, the control group continued to receive normal water, while the high-salinity treatment group was irrigated with artificial saline water containing a 150 mM NaCl l^−1^ solution. Saline water was applied to each subplot for the salinity treatment, using a low-pressure surface irrigation system. The system consisted of a main line (76 mm diameter) delivering saline water from plastic water tanks (5.0 m^3^) to each subplot. The main line was divided into sub-main hoses, each with a manual control valve at every subplot. A similar low-pressure surface irrigation system was used to apply regular water as the control treatment. Irrigation frequency and water application rates were adjusted according to weather conditions and plant growth stages during the wheat growing seasons. To address salt buildup in the root zone under salinity treatment, soil samples were collected from different places within the main plot assigned for the salinity treatment, at a depth of 0–60 cm. Furthermore, all plots in the salinity treatment received three irrigations with normal water before sowing commenced in the second season, to flush out the accumulated salt in the root zone from the first season. The average EC value of all soil samples did not exceed 16.3 dS m^−1^ in both growing seasons. Seeds were manually sown at a rate of 15 g m^−2^ on 25 November 2019 and 17 November 2020, planted at a depth of 3 cm in five 1.5-meter-long rows spaced 0.2 m apart. Fertilization included 100 kg ha^−1^ of P_2_O_5_, 90 kg ha^−1^ of K_2_O, and 150 kg ha^−1^ of N. Phosphorus and potassium were applied before sowing, using calcium superphosphate (18.5% P_2_O_5_) and potassium chloride (50% K_2_O), respectively. Nitrogen was applied in three equal doses at sowing, in the middle of tillering, and at the booting growth stage, using ammonium nitrate (33.5% N). Other agronomic practices, such as weed and disease control, were carried out as recommended. Harvesting of all genotypes took place in the third week of April in both growing seasons.

### 2.3. Measurements

#### 2.3.1. Ion Content

The concentrations of Na^+^, K^+^, and Ca^2+^ ions were measured in dried plant samples collected at the harvest stage (Zodak scale 92) [44], then finely ground with a mill grinder. To prepare the samples for analysis, 0.4 g of finely ground and dried plant samples were digested overnight in digesting tubes containing 8 mL of concentrated nitric acid (HNO_3_) and 3 mL of perchloric acid (HClO_4_). The mixture was then heated at 300 °C for 3 h. After digestion, the solution was cooled, transferred to a 50-mL volumetric flask, and adjusted with distilled water to a final volume of 50 mL. The concentrations of Na^+^, K^+^, and Ca^2+^ were measured using a flame spectrophotometer (ELEX 6361, Eppendorf AG, Hamburg, Germany), and the K^+^/Na^+^ and Na^+^/Ca^2+^ ratios were calculated according to the work of Motsara and Roy [45].

#### 2.3.2. Chlorophyll Content, Leaf Chlorophyll Fluorescence, and Relative Water Content

At the booting stage (Zodak scale 49), 75 days after sowing [44], the contents of different photosynthetic pigments, maximal PSII photochemical efficiency (F_v_/F_m_), and relative water content (RWC) were measured. Photosynthetic pigments were measured using spectrophotometry. Fresh leaf samples (0.4 g) were collected from the uppermost fully expanded leaves and placed in 5 mL of 80% acetone in the dark until the leaves bleached completely. The extracted solution was then centrifuged at 400 rpm for 5 min and adjusted to a final volume of 15 mL with 80% acetone. The optical densities of the extract were measured at 645 nm (A_645_) and 663 nm (A_663_), using a Cary 50 UV-visible spectrophotometer (UV-2550, Shimadzu, Tokyo, Japan). Chlorophyll-a (Chl a), chlorophyll-b (Chl b), and total chlorophyll (Chlt) contents were calculated in mg per g of fresh weight (mg g^−1^ FW^−1^), using the formula developed by Lichtenthaler and Wellburn [46]:

(1) $$Chla=(12.72\times A_{663})-(2.69\times A_{645})\times V/1000\times FW$$

(2) $$Chlb=(22.87\times A_{645})-(4.67\times A_{663})\times V/1000\times FW$$

(3) $$Chlt=(8.02\times A_{663})+(20.21\times A_{645})\times V/1000\times FW$$

where V and FW represent the final volume of the extract solution (15 mL) and the weight of the fresh leaf tissue extracted (0.4 g), respectively.

The maximal PSII photochemical efficiency (F_v_/F_m_) was measured using a portable chlorophyll fluorometer (Photon Systems Instruments, PAM-2100, Walz, Germany). Four plants from each genotype and replicates in the control and salinity treatment groups were randomly selected for measurements, with the second fully expanded leaf from the top of each plant used for this measurement. The selected leaves were dark-adapted for 30 min with light exclusion clips to measure the minimum fluorescence (F_0_). F_0_ was measured with low-intensity modulated light (<0.1 mmol m^−2^ s^−1^) to avoid variable fluorescence. The maximum fluorescence (F_m_) was measured with a saturating light (8000 mmol m^−2^ s^−1^) for 0.8 s on a dark-adapted leaf. The F_v_/F_m_ ratio in the dark-adapted state was calculated using the following formula:

(4) $$F_{v}/F_{m}=(F_{m}-F_{o})/F_{m}$$

To measure relative water content (RWC), leaf samples of approximately 0.20 cm^2^ were collected from the top of four randomly selected plants in both the control and salinity treatment groups. The fresh weight (FW) of the samples was recorded, followed by soaking them in distilled water for 24 h to determine their turgid weight (TW). The samples were then dried at 75 °C until a constant weight was achieved, to determine their dry weight (DW). RWC was calculated using the following formula:

(5) RWC=(FW−DW)/(TW−DW)×100

Canopy temperature was measured for each genotype and replicate in both the control and salinity treatments, using a handheld infrared thermal camera (Therma CAM SC 3000, FLIR System, San Jose, CA, USA) between 10:00 and 13:00. The camera had a thermal sensitivity of ≤0.05 °C at 30 °C and operated in a wavelength range of 7.5–14 µm. It had a 320 × 240-pixel micro-bolometer with a 23° × 17° lens field of view. The canopy emissivity was set to 0.95 for the wet reference and target leaves and 0.96 for the dry reference. The camera was positioned 1.0 m above the plant canopy. Canopy temperature was extracted from the IR image using SmartView Fluke IR imaging software (version 3.2, Fluke Corporation, Plymouth, MN, USA) by averaging the temperature within a polygon area fitted around the selected canopy area.

#### 2.3.3. Growth and Yield

At the booting stage (Zodak scale 49), 10 plants from each genotype and replicate in the control and salinity treatment groups were randomly selected. Their green leaves were separated and measured for green leaf area (GLA) using a leaf area meter (LI 3100; LI-COR Inc., Lincoln, NE, USA). Additionally, the plant dry weight (PDW) was determined by oven-drying all parts of the same 10 plants at 75 °C until a constant weight was achieved.

At the maturity stage (approximately 150 days after sowing), 20 spikes were randomly selected from each genotype and replicate in the control and salinity treatment groups to record grain number per spike (GNPS). Additionally, three inner rows (0.75 m^2^ total area) were hand-harvested, air-dried for 7 days, weighed to determine their biological yield (BY), and then threshed to obtain the grain. The grain was cleaned, adjusted to a moisture content of 14.0%, and weighed to determine the grain yield (GY). The BY and GY were converted to tons per hectare, and the harvest index (HI) was calculated by dividing GY by BY.

### 2.4. Statistical Analysis

The data for ionic, physiological, growth, and yield traits were first checked for normality to identify and remove any outliers. Following this, a Bartlett test was conducted to test the homogeneity of variance for each trait, to assess whether the results from both seasons could be combined for analysis. The impact of the year (Y), salinity (S), genotype (G), and their interactions on each trait were assessed through an analysis of variance (ANOVA). The differences among the mean values of genotypes for each trait under control or salinity conditions were compared using Tukey’s HSD post hoc test at a 5% probability level. Pearson correlation analyses were used to investigate the associations among all traits under controlled or salinity conditions. To simplify and analyze the data, identify the correlations among different traits, and determine the key factors influencing variability in the tested wheat genotypes, principal component analysis (PCA) was performed on the genotype-by-parameter matrix of means. Following this analysis, a biplot was created using the XLSTAT software (vers. 2022.1, Excel add-ins soft SARL, New York, NY, USA). To show the relationships between traits and genotypes and to categorize genotypes based on their salt tolerance levels, a heatmap cluster analysis was conducted for traits under both control and salinity stress conditions. The heatmap was generated using R statistical software (version 4.2.2), with data from both years combined.

## 3. Results

### 3.1. Analysis of Variance

The impacts of salinity treatment (ST), genotypes (G), and their interaction in each year, as well as the impact of the year (Y) and its interaction with ST and G in the two years combined, on various ionic and agro-physiological traits are presented in Table 1. The ANOVA results showed significant differences (*p* ≤ 0.05 and 0.001) between ST and G for all ionic and agro-physiological traits in each year and in the combined two years, except for K^+^ content in the first year and the combined two years and HI in the second year, which did not show significant differences between ST values. Additionally, RWC in the second year did not display significant differences between G values. The interaction between ST and G also significantly affected all agro-morphological traits, except for GNPS in the first year and RWC in the second year (Table 1). Among the ion contents and their ratios, only the K^+^ showed significant differences (*p* ≤ 0.05) between the two years. Different physiological traits like canopy temperature (CT), maximum quantum PSII photochemical efficiency (F_v_/F_m_), chlorophyll a/b ratio (Chla/Chlb ratio), and total chlorophyll content (Chlt) showed significant differences (*p* ≤ 0.05 and 0.01) between Y values, with the exception of RWC. Significant differences (*p* ≤ 0.05) were observed between the years for plant dry weight (PDW), grain number per spike (GNPS), and biological yield (BY) (Table 1). The interaction between G and Y had a significant impact on all agro-morphological traits, with the exception of RWC and yield traits (GNPS, GY, BY, and HI). The interaction between ST and Y did not have a significant effect on all growth and yield traits, but did have a significant effect on all physiological traits, except for the Chla/Chlb ratio, as well as on ionic traits, except for Na^+^ content and K^+^/Na^+^ ratio. The three-way interaction (G × ST × Y) did not show a significant impact on all growth and yield traits, except for GLA, but did have a significant effect on all ionic and physiological traits, except for RWC (Table 1).

### 3.2. Genotypic Performance Under Control and Salinity Conditions Based on Ionic Traits

Under salinity stress, there was a significant increase in Na^+^ content and a slight increase in K^+^ and Ca^2+^ contents in the shoots of cultivars/RILs, resulting in a significant decrease in K^+^/Na^+^ and Ca^2+^/Na^+^ ratios. Over the two-year period, Na^+^ content increased by 1781.2%, K^+^ by 0.84%, and Ca^2+^ by 15.0%. However, the K^+^/Na^+^ ratio decreased by 94.5% and the Ca^2+^/Na^+^ ratio decreased by 93.8% compared to control conditions (Table 2). Additionally, the cultivars/RILs showed significant variations in their performance for ionic traits under both control and salinity stress conditions. For example, over the two-year period, the average values of Na^+^, K^+^, Ca^2+^, K^+^/Na^+^, and Ca^2+^/Na^+^ among the cultivars/RILs ranged from 68.69 to 141.52 mmol kg^−1^ DW, 639.5 to 1245.6 mmol kg^−1^ DW, 400.5 to 497.5 mmol kg^−1^ DW, 6.88 to 14.14, and 3.38 to 6.33, respectively, under control conditions. Under salinity stress, these values ranged from 1404.6 to 2279.9 mmol kg^−1^ DW, 719.5 to 1287.3 mmol kg^−1^ DW, 446.0 to 588.8 mmol kg^−1^ DW, 0.32 to 0.85, and 0.20 to 0.39, respectively (Table 2).

Additionally, the different varieties/RILs exhibited Na^+^ contents in the shoots that were 15 to 23 times higher under salinity stress compared to the control. Conversely, the K^+^/Na^+^ and Ca^2+^/Na^+^ ratios were 10 to 40 times and 11 to 24 times higher, respectively, in the control compared to salinity stress conditions. However, the performance of varieties/RILs in terms of K^+^ and Ca^2+^ contents varied and fluctuated between the salinity stress and control treatments. Some varieties/RILs showed higher values for K^+^ and Ca^2+^ contents under control conditions compared to salinity stress, while others exhibited the opposite trend. Interestingly, the salt-tolerant genotype Sakha 93 and most RILs had higher Ca^2+^ contents under salinity stress than under control conditions, whereas the salt-sensitive genotype Sakha 61 showed the opposite pattern. The salt-tolerant Sakha 93 and salt-sensitive Sakha 61 genotypes, along with most of the RILs produced from their cross (RIL1-group), and the moderately salt-tolerant Sids 1 genotype all showed higher K^+^ contents under normal conditions compared to salinity stress. Conversely, the majority of RILs resulting from the cross between salt-tolerant and moderately salt-tolerant genotypes (RIL2-group) exhibited the opposite trend. Furthermore, the Sakha 93 and most of the RIL2-group showed improved performance under salinity stress by exhibiting low Na^+^ content, higher K^+^ and Ca^2+^ contents, and increased K^+^/Na^+^ and Ca^2+^/Na^+^ ratios, whereas the Sakha 61, Sids 1, and some RIL1-group specimens displayed the opposite pattern (Table 2).

### 3.3. Genotypic Performance Under Control and Salinity Conditions, Based on Physiological Traits

Compared to the control treatment, salinity stress led to a decrease of 17.8% in RWC, 4.1% in F_v_/F_m_, 15.2% in the Chla/Chlb ratio, and 28.1% in Chlt, while CT increased by 34.8% (Table 3). Furthermore, there were notable variations in the physiological traits among varieties/RILs under either normal or salinity stress treatments. For example, the RWC ranged from 76.28% to 83.98% and from 62.12% to 69.69%, CT ranged from 25.76 °C to 28.37 °C and from 33.39 °C to 39.06 °C, F_v_/F_m_ ranged from 0.79 to 0.83 and from 0.72 to 0.84, the Chla/Chlb ratio ranged from 1.67 to 4.73 and from 1.31 to 2.98, and Chlt ranged from 3.57 mg g^−1^ FW to 4.89 mg g^−1^ FW and from 2.13 mg g^−1^ FW to 3.75 mg g^−1^ FW under control and salinity stress conditions, respectively. Interestingly, the varieties/RILs showed varied responses when comparing the mean values of different physiological traits under control and salinity stress conditions.

Although the RWC decreased and CT increased due to salinity stress in all varieties/RILs, the degree of decrease or increase differed among the varieties/RILs. The decrease in RWC and increase in CT percentages for the salt-tolerant Sakha 93 (13.5% and 31.9%) and moderately salt-tolerant Sids 1 (12.7% and 31.6%) were less than those for the salt-sensitive Sakha 61 (21.5% and 39.5%). Additionally, the decrease in RWC (17.0–23.3%) and increase in CT (28.4–49.0%) for several RILs from the RIL1-group under salinity stress were higher or similar to those observed in the salt-sensitive Sakha 61. In contrast, the decrease in RWC and increase in CT for several RILs from the RIL2-group under salinity stress were lower or comparable to those seen in the salt-tolerant Sakha 93 (Table 3). Similarly, the percentage decrease in Chlt varied among the genotypes in response to salinity stress. The reduction in Chlt was lower for the salt-tolerant Sakha 93 (21.9%) and moderately salt-tolerant Sids 1 (31.9%) compared to the salt-sensitive Sakha 61 (40.8%). The decrease in Chlt due to salinity stress seen in several RILs in the RIL2-group under salinity stress was comparable to the salt-tolerant Sakha 93. Conversely, the decrease in Chlt seen in several RILs in the RIL1-group, except for RIL1-6, was similar to that in the salt-sensitive Sakha 61 (Table 3). The response of the F_v_/F_m_ and Chla/Chlb ratios to salinity stress was not consistent among the varieties/RILs. For example, the F_v_/F_m_ ratio increased in the salt-tolerant Sakha 93 and RIL1-6 and remained unchanged in RIL1-7, RIL2-4, and RIL2-6 in response to salinity stress. In contrast, it decreased by 3.2% in the moderately salt-tolerant Sids 1, by 11.2% in the salt-sensitive Sakha 61, and by 3.0% to 9.4% in the remaining RILs from the RIL1-group, as well as by 2.1% to 9.3% in the remaining RILs from the RIL2-group (Table 3). Similarly, the Chla/Chlb ratio significantly increased in Sakha 93, RIL1-6, RIL1-2, RIL2-4, RIL2-5, RIL2-7, RIL2-8, and RIL2-11 (12.6–60.2%) under salinity stress, while it significantly decreased in Sakha 61 (72.3%) and the other RILs from the RIL1-group (35.2–57.2%). However, there was a moderate decrease in the Chla/Chlb ratio in Sids 1 (17.0%) and the other RILs from the RIL2-group (9.3–18.3%) in response to salinity stress (Table 3).

### 3.4. Genotypic Performance Under Control and Salinity Conditions, Based on Growth and Yield Traits

Under salinity stress at 150 mM NaCl, there was a reduction in PDW by 26.4%, GLA by 39.9%, GNPS by 23.1%, GY by 33.5%, BY by 24.1%, and HI by 11.3% compared to the control (Table 4). Additionally, there were significant differences in these traits among various varieties/RILs under both normal and salinity stress conditions. For instance, the PDW ranged from 5.02 g plant^−1^ to 6.99 g plant^−1^ and from 3.36 g plant^−1^ to 5.40 g plant^−1^, GLA ranged from 142.7 cm^2^ plant^−1^ to 323.7 cm^2^ plant^−1^ and from 106.5 cm^2^ plant^−1^ to 157.9 cm^2^ plant^−1^, GNPS ranged from 45.9 to 55.8 and from 33.3 to 44.4, GY ranged from 497.7 kg m^2^ to 678.4 kg m^2^ and from 304.1 kg m^2^ to 457.7 kg m^2^, BY ranged from 1509.4 kg m^2^ to 2171.5 kg m^2^ and from 943.1 kg m^2^ to 1630.1 kg m^2^, and HI ranged from 29.4% to 37.4% and from 24.1% to 37.0% under control and salinity stress conditions, respectively (Table 4). Furthermore, the varieties/RILs exhibited varying responses in terms of mean values for different growth and yield traits under salinity stress conditions. On average, there was a decrease of 6.4% to 47%, 5.9% to 60.0%, 15.9% to 32.6%, 17.3% to 47.3%, and 1.3% to 52.6% in PDW, GLA, GNPS, GY, and BY, respectively, under salinity stress compared to the control. The reduction percentages in the different growth and yield traits were higher for the salt-sensitive Sakha 61 compared to the salt-tolerant Sakha 93 and moderately salt-tolerant Sids 1. Additionally, the reduction percentages in the different growth and yield traits for several RILs from the RIL1-group due to salinity stress were higher than those observed in the salt-sensitive Sakha 61. In contrast, the reduction percentages in different growth and yield traits for several RILs from the RIL2-group due to salinity stress were lower than those seen in the salt-tolerant Sakha 93 (Table 4). For HI, the salt-tolerant Sakha 93 and three RILs (RIL1-1, RIL1-4, and RIL2-10) exhibited an increase in HI as a result of salinity stress. The moderately salt-tolerant Sids 1 and the salt-sensitive Sakha 61 showed the highest reduction percentages for HI, with salinity stress decreasing HI by 20.3% and 20.8%, respectively, compared to the control. Salinity stress caused a notable decrease in HI in several RILs from two groups, with reductions ranging from 9.20% to 23.0% (Table 4).

### 3.5. Correlation Matrix Between All Traits Under Control and Salinity Stress Conditions

**Figure 1:**
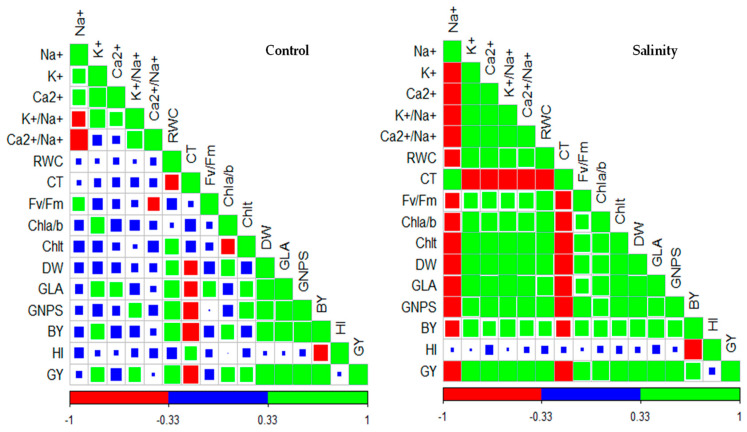
Pearson’s correlation coefficients among different ionic, physiological, growth, yield, and yield component traits of wheat genotypes grown under control and salinity stress conditions. Na^+^, K^+^, and Ca^2+^: contents of sodium, potassium, and calcium (mmol kg^−1^ DW), respectively; RWC: relative water content (%); CT: canopy temperature (°C); Fv/Fm: maximum quantum PSII photochemical efficiency; Chla/Chlb: chlorophyll a/b ratio; Chlt: total chlorophyll content (mg g^−1^ FW); PDW: plant dry weight (g plant^−1^); GLA: green leaf area (cm^2^ plant^−1^); GNPS: grain number per spike; BY: biological yield (ton m^2^); GY: grain yield (ton m^2^); and HI: harvest index (%).

Overall, the various agro-physiological traits exhibited stronger correlations with each other in salinity stress conditions than in control conditions (Figure 1). All ionic and physiological traits showed a strong correlation with growth traits (PDW and GLA), GNPS, and GY and a moderate correlation with BY under salinity stress conditions. However, under control conditions, only K^+^ content, RWC, CT, and Chlt showed a moderate correlation with growth traits and GY, while the other ionic and physiological traits did not (Figure 1). Under salinity stress, there was a strong correlation between all ionic traits and physiological traits, except for F_v_/F_m_, which showed a moderate correlation with ionic traits. In contrast, there were no significant correlations found between ionic and physiological traits under normal conditions (Figure 1). Both growth traits (PDW and GLA) and GNPS exhibited a strong correlation with GY and BY under control and salinity stress conditions. There was no correlation between any traits and HI in both control and salinity stress conditions, except for BY, which exhibited a strong negative correlation with HI under salinity stress (r = −0.78) and a moderately negative correlation under control (r = −0.52) conditions (Figure 1).

### 3.6. Comprehensive Evaluation of Salt Tolerance in Genotypes Through Multivariate Analysis

#### 3.6.1. Principal Component Analysis

**Figure 2:**
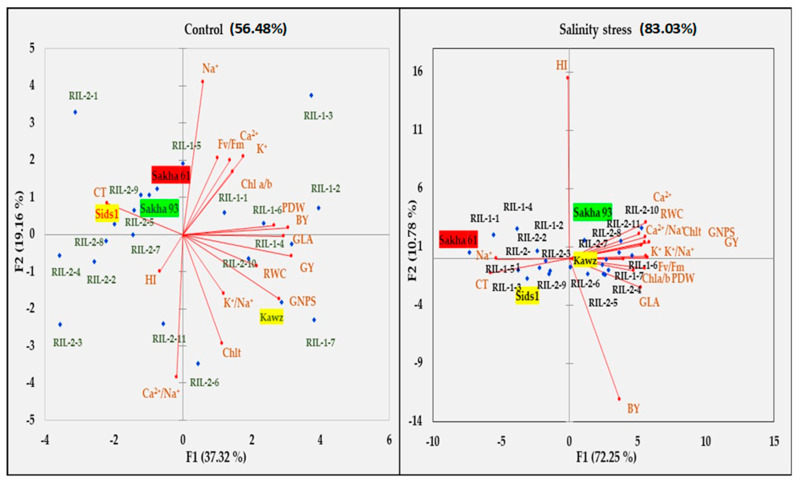
Principal component analysis biplot showing the relationship between genotypes and traits under both control and salinity conditions. Na^+^, K^+^, and Ca^2+^: contents of sodium, potassium, and calcium (mmol kg^−1^ DW), respectively; RWC: relative water content (%); CT: canopy temperature (°C); Fv/Fm: maximum quantum PSII photochemical efficiency; Chla/Chlb: chlorophyll a/b ratio; Chlt: total chlorophyll content (mg g^−1^ FW); PDW: plant dry weight (g plant^−1^); GLA: green leaf area (cm^2^ plant^−1^); GNPS: grain number per spike; BY: biological yield (ton m^2^); GY: grain yield (ton m^2^); and HI: harvest index (%). The red, yellow, and green shadows correspond to the salt-sensitive, moderately salt-tolerant, and salt-tolerant genotypes, respectively.

Principal component analysis (PCA) was performed to illustrate the relationships between all traits, identify the key traits affecting the response to salinity stress, and assess their ability to distinguish between salt-tolerant and salt-sensitive genotypes under both control and salinity stress conditions (Figure 2 and Table 5). Under control conditions, the first five principal components (PCs) had eigenvalues greater than 1 and accounted for 85.59% of the total variation among all variables (traits and genotypes). The first two PCs accounted for 56.48% of the total variability, with PC1 explaining 37.32%. PC1 exhibited strong positive correlations with growth traits (PDW and GLA), GY, and yield components (GNPS and BY), as well as moderate positive correlations with RWC traits. Additionally, PC1 showed a moderate negative correlation with CT. PC2 represented 19.16% of the total variability and had a strong positive correlation with Na^+^ content, a strong negative correlation with Ca^2+^/Na^+^, a moderate positive correlation with F_v_/F_m_, and a moderate negative correlation with Chlt. PC3, accounting for 14.07% of the total variability, exhibited a strong positive correlation with K^+^/Na^+^ and a moderate positive correlation with K^+^ and Ca^2+^ contents. PC5, only representing 6.56% of the total variability, showed a moderate positive correlation with the Cha/Chlb ratio (Table 5). Under salinity stress conditions, the first two PCs accounted for the majority of variation among all variables (83.04%), with PC1 explaining 72.25% and PC2 explaining 10.78% of the total variability, respectively. PC1 exhibited strong negative correlations with Na^+^ content and CT, as well as strong positive correlations with other ionic, physiological, growth, and yield traits, with the exception of BY and HI, which displayed strong negative and positive correlations with PC2, respectively (Table 5 and Figure 2).

Based on the PCA biplot, the various varieties/RILs were spread out in all four quadrants of the biplot in control conditions, whereas most of them were grouped together in only two quadrants under salinity stress conditions (Figure 2). The salt-tolerant Sakha 93 and salt-sensitive Sakha 61 were located in the low PC1 and high PC2 regions of the biplot in control conditions. However, under salinity stress conditions, Sakha 93 shifted to high PC1 and PC2, while Sakha 61 moved to low PC1 and high PC2 (Figure 2). Importantly, the PCA biplot clearly separated the traits into two distinct groups under salinity stress conditions. The first group encompassed almost all traits, while the second group comprised Na^+^ content, CT, BY, and HI. The traits in the first group displayed significant positive correlations with each other, whereas the traits in the second group demonstrated marked negative correlations, both within the group and with the traits in the first group. Salt-tolerant Sakha 93 and Kawz, along with two RILs from the RIL1-group and seven RILs from the RIL2-group, showed close associations with traits in the first group, while the salt-sensitive Sakha 61, Sids 1, five RILs from the RIL1-group, and three RILs from the RIL2-group were closely associated with traits in the second group (Figure 2).

#### 3.6.2. Heatmap Cluster Analysis

**Figure 3:**
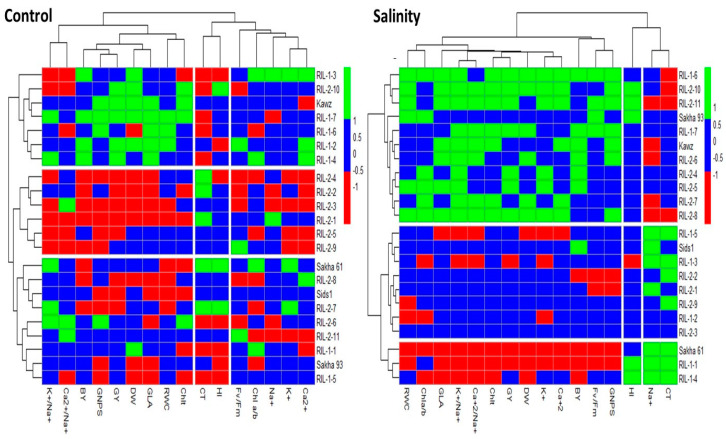
Clustering heatmap showing the performance of 22 RILs/varieties under control and salinity stress conditions, based on all measured traits. Na^+^, K^+^, and Ca^2+^: contents of sodium, potassium, and calcium (mmol kg^−1^ DW), respectively; RWC: relative water content (%); CT: canopy temperature (°C); Fv/Fm: maximum quantum PSII photochemical efficiency; Chla/Chlb: chlorophyll a/b ratio; Chlt: total chlorophyll content (mg g^−1^ FW); PDW: plant dry weight (g plant^−1^); GLA: green leaf area (cm^2^ plant^−1^); GNPS: grain number per spike; BY: biological yield (ton m^2^); GY: grain yield (ton m^2^); and HI: harvest index (%).

Two-way clustering heatmaps were utilized to classify the varieties/RILs into different groups based on all traits (Figure 3). The heatmap divided genotypes into three groups in both conditions. It effectively distinguished the salt-sensitive Sakha 61 from the salt-tolerant Sakha 93 under salinity stress conditions, while grouping both genotypes together under control conditions. The group containing the two genotypes (Sakha 93 and Sakha 61) under control conditions did not show the highest values for K^+^ and Ca^2+^ contents, RWC, Chlt, growth, and yield traits. Rather, the values of these traits for this group were intermediate between the values of the genotypes in the first and second groups. Additionally, the group with the two genotypes showed higher values for K^+^/Na^+^ and Ca^2+^/Na^+^ ratios, CT, and HI (Table 6). However, under salinity stress conditions, the group with Sakha 93 exhibited higher values for all traits except Na+ content, CT, and HI, compared to the group with Sakha 61. The group with Sakha 93 showed a 36.9% decrease in Na^+^ content, a 10.0% decrease in CT, and a 16.7% decrease in HI compared to the group with Sakha 61. Additionally, the group with Sakha 93 displayed a 6.3–51.4% increase in other ionic, physiological, growth, and yield traits compared to the group with Sakha 61 (Table 6).

## 4. Discussion

Developing salt-tolerant wheat varieties and providing them to farmers is considered a cost-effective solution to address salinity issues. Despite extensive efforts over the last two decades, the development of wheat genotypes that are tolerant to salt stress and can yield economically under salinity stress conditions remains a significant challenge. This challenge is primarily due to the fact that most salinity experiments are conducted in controlled environments rather than in real field conditions, where genotypes are exposed to the complex interactions of various environmental factors. Additionally, there is a lack of reliable screening criteria for assessing the salt tolerance of genotypes in field conditions and restricted genetic diversity regarding salt tolerance, as well as a lack of experiments that evaluate salt tolerance at different growth stages, not just during the germination and seedling stages [1,11,14,18]. Therefore, it is essential to gain a comprehensive understanding of how various plant traits react to salinity stress in different wheat genotypes at various growth stages in real field conditions. This in-depth understanding will be vital for the success of breeding programs aimed at improving the salt tolerance of genotypes. This study evaluated the salt tolerance of 22 bread wheat genotypes, using the salt-sensitive Sakha 61, the salt-tolerant Sakha 93, and the moderately salt-tolerant Sids 1 genotypes as benchmarks for salt tolerance. This study also involved 7 F_8_ RILs derived from crossing the first and second genotypes (RIL1-group) and 11 RILs derived from crossing the second and third genotypes (RIL2-group). The evaluation took place during different growth stages in real field conditions, with a focus on various ionic and agro-physiological traits. The ANOVA results indicate that different ionic and agro-physiological traits had significant responses to salinity stress and showed significant variations among genotypes when the data were analyzed for individual years and combined over two years (Table 1). These findings indicate that the ionic and agro-physiological traits measured at different growth stages can serve as reliable screening criteria for evaluating the salt tolerance of genotypes in real field conditions, which will be discussed below in more detail.

### 4.1. Ionic Traits as Screening Criteria

Studies on ionic content in plants have demonstrated that salt-tolerant genotypes can mitigate the detrimental impacts of Na^+^ toxicity by keeping a low Na^+^ content while maintaining high contents of essential ions like K^+^ and Ca^2+^ in their shoots. This mechanism ultimately keeps the ratios of K^+^/Na^+^ and Ca^2+^/Na^+^ at high levels compared to salt-sensitive genotypes [11,47,48,49]. In general, elevated levels of NaCl in the root zone of plants usually lead to higher uptake of Na^+^ by the root cells via transport mechanisms and active channels, causing an increased accumulation of Na^+^ in the leaves to toxic levels. This disrupts the balance of cations and increases the production of reactive oxygen species (ROS), which can damage leaves, reduce chlorophyll production and photosynthesis efficiency, and alter enzyme functions. Ultimately, this can lead to inhibited plant growth and decreased yields [11,23,50]. Additionally, due to the similar chemical properties of Na^+^ and K^+^ in terms of ionic radius and hydration energy, salt tolerance necessitates not only a restricted Na^+^ intake but also the uptake of K^+^, which is vital for numerous important plant functions such as photosynthesis, osmoregulation, chlorophyll pigment biosynthesis, and the activation of more than 50 enzymes [2,51,52,53]. Furthermore, the interplay between Na^+^ and Ca^2+^ is essential in determining plant tolerance to Na^+^ toxicity since an excess of Na^+^ can interfere with Ca^2+^ functions and hinder the binding sites of Ca^2+^ in plant organs [2,54]. Ca^2+^ also plays a role in mitigating the adverse impacts of salinity stress by enhancing K^+^/Na^+^ selectivity, acting as a secondary messenger in plant signaling, and protecting the integrity of the plasma membrane [55]. Therefore, previous studies have shown that the selective uptake of K^+^ and Ca^2+^ over Na^+^ and the maintenance of high intracellular K^+^/Na^+^ and Ca^2+^/Na^+^ ratios under salinity stress conditions are essential physiological mechanisms that contribute to salt tolerance in genotypes in several important field crops like wheat [2,8,11,17,20,21]. This highlights the significance of Na^+^, K^+^, and Ca^2+^ contents and their ratios as reliable and effective screening criteria for evaluating salt tolerance in genotypes under real field conditions. In this study, the Na^+^ content in all varieties/RILs increased significantly by 1781.2% under salinity stress, with values ranging from 1404.6 to 2279.9 mmol kg^−1^ DW among genotypes. However, K^+^ and Ca^2+^ contents only slightly increased, by 0.84% and 15.0%, respectively, and K^+^/Na^+^ and Ca^2+^/Na^+^ ratios significantly decreased by about 94% under salinity stress, with values ranging from 719.5 to 1287.3 mmol kg^−1^ DW for K^+^, 446.0 to 588.8 mmol kg^−1^ DW for Ca^2+^, 0.32 to 0.85 for K^+^/Na^+^, and 0.20 to 0.39 for Ca^2+^/Na^+^ (Table 2). This wide range of ion contents among varieties/RILs under salinity stress conditions makes these traits an effective and reliable screening tool for differentiating between salt-tolerant and salt-sensitive wheat genotypes. This is evident when comparing the ion contents and their ratios in the salt-tolerant reference genotype Sakha 93 and the salt-sensitive reference genotype Sakha 61. Under salinity stress, Sakha 93 showed a 23.8% decrease in Na^+^ content compared to Sakha 61. However, it also showed higher K^+^ and Ca^2+^ contents, as well as K^+^/Na^+^ and Ca^2+^/Na^+^ ratios by 30.1%, 15.7%, 72.2%, and 51.5%, respectively, compared to Sakha 61. Additionally, most RILs from the RIL2-group also accumulated less Na^+^ and maintained slightly higher or similar levels of K^+^ and Ca^2+^ compared to the control (Table 2). These findings also confirm that the different ionic traits measured under salinity stress conditions may be reliable screening criteria for evaluating salt tolerance in wheat genotypes grown in real field conditions. This is further supported by the strong correlations observed between various ionic traits and the growth, yield, and yield components under salinity stress, while these correlations were weak or insignificant under control conditions (Figure 1). This indicates that the genotypic variation in growth and yield under salinity stress is closely related to the genotypic variation in ion contents. Therefore, it can be concluded that detecting the ion contents of genotypes under salinity stress conditions is crucial as a screening criterion to differentiate between salt-tolerant and salt-sensitive wheat genotypes, with the salt-tolerant genotypes usually tending to selectively take up K^+^ and Ca^2+^ over Na^+^ in order to maintain a higher K^+^/Na^+^ and Ca^2+^/Na^+^ ratio, while the salt-sensitive genotypes do not. This is consistent with the findings from several previous studies on different crops [2,47,50,56].

### 4.2. Physiological Traits as Screening Criteria

The osmotic phase of salinity stress, caused by the reduced osmotic potential of the soil solution, is well known for hindering a plant’s water absorption capacity, similar to water deficit stress. This impacts the leaf’s water balance, especially its RWC, which reflects the balance between water uptake and transpiration rate. The decrease in RWC and K^+^ content due to salinity stress causes stomatal closure and a notable decrease in transpiration rate, which is essential for cooling the canopy. As a result, an increase in canopy surface temperature becomes a common phenomenon when plants are exposed to salinity stress, even for a short period [29,30,57]. Additionally, the combination of stomatal closure, reduced leaf RWC, and increased CT, along with the buildup of toxic levels of Na^+^ in leaves, results in an excess production of ROS. These ROS ultimately inhibit the function of the photosystem II complex (PSII) on both the acceptor and donor sides and also lead to the degradation of chlorophyll pigments by either reducing chlorophyll synthesis or accelerating chlorophyll breakdown [8,47,58]. These close interconnections between these physiological processes suggest that traits like RWC (indicating overall plant water content), CT (related to canopy cooling), F_v_/F_m_ (measuring the maximum efficiency of PSII in converting absorbed light into chemical energy), and photosynthetic pigments (involved in absorbing longwave light (Chla) and shortwave light (Chlb), electron transport, and energy conversion) may be valuable selection criteria for assessing salt tolerance in different genotypes and identifying potential genetic sources of salt tolerance. In this study, exposure to high salinity led to a slight decrease in F_v_/F_m_ (4.1%) and a moderate reduction in the RWC (17.8%) and Cha/Chlb ratios (15.2%), as well as a significant decrease in Chlt (28.1%) and an increase in CT (34.8%) compared to the control treatment (Table 3). These findings indicate that assessing CT, RWC, and photosynthetic pigments together is crucial for evaluating salt tolerance in wheat genotypes under real field conditions, while the reliability of Fv/Fm in such conditions is limited and may vary, depending on the genotype. Previous studies have shown that RWC and Chlt display significant differences among the genotypes examined, with a reduction of 15–20% observed under salinity stress compared to the control treatment [2,34,48,56,59,60]. However, the decrease in RWC and Chlt was generally less pronounced in the salt-tolerant germplasm compared to salt-sensitive varieties. For instance, Quan et al. [9] found that the reduction in Chlt in moderately salt-tolerant wheat germplasm ranged from 10–16%, while it exceeded 20% in the salt-sensitive varieties. Additionally, Amirjani [61] also reported that treating rice crops with 200 mM NaCl resulted in a significant decrease in Chla and Chlb, with Chlb (41.0%) showing a higher decrease compared to Chla (33.0%). As a result, RWC and chlorophyll pigment contents (Chla, Chlb, and Chlt) are commonly used as reliable screening criteria for evaluating salt tolerance in various germplasm materials. The smaller decrease in Chlt for the salt-tolerant genotype Sakha 93 (21.9%) compared to the salt-sensitive genotype Sakha 61 (40.8%) may be due to the salt-tolerant genotype’s ability to exclude Na^+^ from the leaves and maintain higher levels of K^+^ and Ca^2+^ in the leaves compared to the salt-sensitive genotype. The accumulation of excess Na^+^ in the leaves can lead to the production of ROS, which can disrupt the pigment biosynthesis pathways and damage chloroplast membranes [34,62]. This finding is supported by the strong correlation observed between ionic traits and the Chla/Chlb ratio and Chlt, particularly under conditions of salinity stress (Figure 1).

Previous research has also found that thermal imaging can effectively distinguish between different genotypes grown under normal conditions or under salinity stress, with the average CT values of genotypes grown in salinity stress conditions being approximately 5 °C higher than those grown under normal conditions [63]. In this research, exposure to salinity stress resulted in a 31.9% increase in CT for the salt-tolerant genotype Sakha 93 and a 39.5% increase for the salt-sensitive genotype Sakha 61. Furthermore, some RILs from the RIL2-group exhibited lower CT values than Sakha 93, while some RILs from the RIL1-group showed higher CT values than Sakha 61 under salinity stress (Table 3). This result indicates that it is possible to evaluate salt tolerance in wheat genotypes using thermal image analysis. The rise in CT of several varieties/RILs under salinity stress may be attributed to the osmotic stress caused by salinity, which affects water absorption and reduces the RWC of leaves. Additionally, the build-up of excessive Na^+^ and decreased K^+^ levels in leaves can result in stomatal closure. This ultimately leads to a decrease in transpiration rate, which is crucial for leaf cooling and lowering the plant’s temperature [29,30,64]. This finding is supported by the strong correlation that was observed between ionic traits and RWC with CT, particularly under salinity stress conditions (Figure 1). Our results suggest that thermal imaging can be a quick, non-destructive, and cost-effective method for high-throughput phenotyping to evaluate the salt tolerance of a large number of genotypes.

However, there are conflicting results on the impact of salinity stress on the F_v_/F_m_ ratio. Some studies have found no significant effect of salinity stress on the F_v_/F_m_ ratio, leading to it being considered ineffective as a screening criterion for detecting salt tolerance in genotypes [65,66]. In contrast, other studies have shown a notable decrease in the F_v_/F_m_ ratio under salinity stress, with salt-tolerant genotypes displaying higher F_v_/F_m_ ratios compared to salt-sensitive ones. Therefore, these results suggest that the F_v_/F_m_ ratio can be used as a quick, reliable, and non-invasive method for distinguishing salt-tolerant genotypes from salt-sensitive ones [67,68]. The inconsistent results from studies on F_v_/F_m_ may be attributed to the fact that measuring this trait requires special care or that it is also genotype-dependent. In this study, the F_v_/F_m_ ratio decreased by 11.2% for the salt-sensitive Sakha 61, while it increased by 1.7% for the salt-tolerant Sakha 93 compared to the control. This suggests that while F_v_/F_m_ may not be suitable for assessing salt tolerance in general, it is still a quick, reliable, and non-invasive way to distinguish between salt-tolerant and salt-sensitive genotypes.

### 4.3. Growth and Yield Traits as Screening Criteria

Evaluating salt tolerance in crops, particularly under real field conditions, also requires investigating growth and yield traits, as these traits indicate the adverse effects of salinity stress factors (osmotic stress, ion toxicities, and ion imbalance) at the overall plant level. They also demonstrate the extent of salinity stress effects on the physiological and biochemical processes associated with plant growth, development, and production [2,8,11,49]. For example, GLA reflects the negative impacts of salinity stress factors on cell elongation and division, leaf morphology, and thickness, as well as leaf damage and the ratio of living to dead leaves [23]. Additionally, PDW is another valuable approach for studying the relationship between salinity stress and the associated biochemical and physiological changes at critical growth stages in plants [11]. Most importantly, the final GY and its components are the outcomes of a range of physiological activities and growth processes, such as biomass allocation, plant photosynthetic activity, the availability of photo-assimilates, and the transport of photo-assimilates from source to sink, which occur throughout the different growth stages [14,18,49]. These facts highlight the importance of growth and yield traits as a comprehensive indicator of genotype performance and salt stress tolerance. Thus, assessing these traits can be a valuable screening criterion for identifying salt tolerance among genotypes in real field conditions. In this study, salinity stress led to a significant decrease in various growth parameters (PDW and GLA), yield components (GNPS and BY), and GY traits by 23.1% to 39.9%. The reduction in these traits was approximately twice as much in the salt-sensitive Sakha 61 compared to the salt-tolerant Sakha 93 (Table 4). The reduced impact on growth and yield traits in salt-tolerant genotypes compared to salt-sensitive ones may be attributed to the former’s ability to accumulate lower levels of Na^+^ and higher levels of K^+^ and Ca^2+^ ions and their ratio with Na^+^, as well as their capacity to reduce their CT compared to the latter genotypes. These characteristics enable salt-tolerant genotypes to have fewer damaged leaves, a higher ratio of living to dead leaves, a greater supply of assimilates from leaves to growing grains and spikes during the grain filling stage, and, ultimately, result in the highest PDW and GY values [23,69]. These explanations were further supported by correlation analysis, which revealed a strong correlation between growth and yield traits with ionic and physiological traits under salinity stress conditions. In contrast, these correlations were weak or insignificant under control conditions (Figure 1). The results of the correlation analysis emphasized the importance of growth and yield traits as screening criteria under salinity stress conditions, on the one hand, and underscored the role of ionic accumulation, CT, and RWC in determining the salt tolerance of wheat genotypes in real field conditions on the other hand.

### 4.4. Multivariate Analysis for Comprehensive Evaluation of Salt Tolerance in Genotypes

Due to the complexity of salt tolerance in plants, which is determined by multiple genes and reflects the plant’s genetics, physiology, and biochemistry, a comprehensive and accurate evaluation of salt tolerance in different genotypes should consider a range of indicators rather than relying on a single measure [14,59]. Multivariate statistical analysis, such as PCA and heatmap clustering, is frequently employed to evaluate salt tolerance in genotypes, due to its numerous advantages. These include the ability to assess salt tolerance using multiple traits, eliminating bias from a single trait, considering the interaction between various traits, elucidating complex genotype relationships in a more comprehensible way, and condensing highly correlated traits into a reduced set of variables known as principal components [14,61,70,71]. In this study, the PCA and heatmap clustering successfully differentiated between the salt-tolerant Sakha 93 and the salt-sensitive Sakha 61 under salinity stress conditions (Figure 2 and Figure 3). Furthermore, the group consisting of salt-tolerant genotypes exhibited lower Na^+^ content and CT, along with higher K^+^/Na^+^ and Ca^2+^/Na^+^ ratios, RWC, photosynthetic pigmentation, growth, and yield under salinity stress compared to the salt-sensitive group (Table 6). These findings further support the importance of multiple integrated mechanisms in improving and evaluating the salt tolerance of wheat genotypes in field conditions. This is because of the intricate and interdependent relationships among biological processes in plants, where any disturbance to one biochemical process can affect other physiological processes, and vice versa [72]. For instance, the salt tolerance of genotypes is not only determined by their ability to exclude Na^+^; it also involves a greater affinity for K^+^ and Ca^2+^ compared to Na^+^ during ion uptake [11,48,49]. Therefore, in order to protect the plant from specific-ion toxicity, it is necessary to not only exclude Na^+^ but also to ensure the uptake of K^+^ and Ca^2+^, which are crucial for osmotic adjustment and enhancing plant water status (RWC). Increasing the K^+^ content and RWC enhances stomatal conductance and transpiration rate, which are crucial for cooling the canopy. This, in turn, helps in regulating canopy temperature under salinity stress [57]. Additionally, a low sodium content helps to maintain high potassium and calcium levels, which, in turn, protect against the production of ROS. This protection helps to maintain the function of the photosystem II complex and prevent chlorophyll breakdown [47,58]. The close connections among these processes highlight the importance of using multivariate statistical analysis to comprehensively assess salt tolerance in genotypes. This type of analysis offers a comprehensive view of how different traits are related and which traits can be used as primary, alternative, or supplementary screening criteria for evaluating salt tolerance in genotypes grown in real field conditions.

## 5. Conclusions

The study systematically investigated the salt tolerance of wheat genotypes grown in real field conditions by analyzing various ionic and agro-physiological traits comprehensively. The results indicate that the salt tolerance of genotypes is associated with their ability to absorb low levels of Na^+^ while maintaining high levels of K^+^ and Ca^2+^, leading to increased K^+^/Na^+^ and Ca^2+^/Na^+^ ratios in their shoots. These ionic characteristics are connected to the ability of salt-tolerant genotypes to maintain high RWC and Chlt levels, as well as to regulate CT. Therefore, these ionic and physiological traits could be used regularly as screening criteria for evaluating the salt tolerance of wheat genotypes, particularly those traits that are easily and cost-effectively measured, such as CT. The strong correlation between ionic and physiological traits and the growth and yield traits under salinity stress highlights the importance of these traits as indicators for the salt tolerance of wheat genotypes, particularly since these correlations were weak or insignificant under control conditions. Finally, our findings support the use of multivariate analysis as an effective and practical tool in wheat breeding programs for a comprehensive evaluation of salt tolerance in wheat genotypes under real field conditions. This approach allows for the simultaneous consideration of multiple traits to discriminate genotypes based on their salt tolerance levels.

## Figures and Tables

**Table 1:** Analysis of variance (F values) of various ionic and agro-physiological traits for 22 genotypes (G) under salinity treatment (ST) in each year (Y) and the combined data over two years.

SOV	First Year (2019–2020)			Second Year (2020–2021)			Combined Two Years						
	ST	G	G × ST	ST	G	G × ST	Y	ST	G	G × Y	G × ST	ST × Y	G × ST × Y
Df	1	21	21	1	21	21	1	1	21	1	21	21	21
Na^+^	3553.26 ***	4.76 **	4.26 ***	4516 ***	6.88 ***	6.19 ***	1.49 ^ns^	7989.04 ***	9.42 ***	2.38 **	8.31 ***	3.03 ^ns^	2.26 **
K^+^	8.05 ^ns^	10.51 ***	17.82 ***	6.58 *	7.34 ***	27.21 ***	21.29 *	0.05 ^ns^	14.24 ***	3.27 ***	41.51 ***	14.28 *	4.53 ***
Ca^2+^	212.58 **	15.37 ***	10.69 ***	648.55 **	6.24 ***	20.74 ***	0.02 ^ns^	859.91 ***	15.09 ***	6.78 ***	16.14 ***	193.02 ***	15.00 ***
K^+^/Na^+^	455.25 **	25.33 ***	26.75 ***	1201.27 ***	62.39 ***	60.61 ***	2.28 ^ns^	1364.18 ***	70.91 ***	3.31 ***	72.51 ***	1.87 ^ns^	2.52 ***
Ca^2+^/Na^+^	19,552.76 ***	15.15 ***	14.19 ***	770.30 **	16.37 ***	13.88 ***	1.15 ^ns^	3085.47 ***	28.33 ***	2.96 ***	24.90 ***	1.42 ***	3.22 ***
RWC	6640.32 ***	2.24 **	1.92 **	485.20 **	1.43 ^ns^	0.96 ^ns^	1.09 ^ns^	2419.10 ***	2.67 ***	1.02 ^ns^	2.24 **	62.77 **	0.66 ^ns^
CT	6448.15 ***	11.48 ***	14.35 ***	4530.65 ***	12.73 ***	9.59 ***	703 **	10,899.67 ***	20.17 ***	3.97 ***	20.68 ***	89.78 ***	3.49 ***
FV/FM	625 **	4.17 ***	3.29 ***	1980.25 ***	6.09 ***	4.37 ***	22.94 *	2298.4 ***	5.74 ***	5.34 ***	5.34 ***	99.58 ***	2.78 ***
Chla/Chlb	8.97 ^ns^	3.437 ***	17.29 ***	40.60 *	4.73 ***	19.21 ***	26.96 *	49.28 **	5.58 ***	3.06 ***	31.11 ***	23.95 **	6.08 ***
Chlt	677.14 **	12.23 ***	4.06 ***	1195.12 ***	18.16 ***	1.33 ^ns^	503.58 **	1870.02 ***	27.32 ***	5.07 ***	2.996 ***	110.75 ***	1.46 ^ns^
PDW	793.99 **	8.20 ***	9.24 ***	68.60 *	6.35 ***	4.76 ***	23.44 *	314.28 ***	11.88 ***	2.41 ***	12.06 ****	5.01 ^ns^	1.32 ^ns^
GLA	981.56 ***	7.91 ***	6.38 ***	9785.80 ***	28.23 ***	27.88 ***	0.53 ^ns^	3695.37 ***	24.16 ***	5.06 ***	211.63 ***	1.68 ^ns^	5.31 ***
GNPS	513.82 **	3.76 ***	1.63 ^ns^	5494.42 ***	6.34 ***	2.17 **	32.16 *	2046.01 ***	8.31 ***	1.06 ^ns^	3.12 ***	5.24 ^ns^	0.54 ^ns^
GY	1379.46 ***	9.79 ***	8.99 ***	2615.50 ***	5.19 ***	3.17 ***	11.09 ^ns^	3663.93 ***	11.83 ***	1.47 ^ns^	9.44 ***	2.03 ^ns^	0.57 ^ns^
BY	304.55 **	5.20 ***	5.08 ***	65.80 *	6.16 ***	7.42 ***	41.40 *	225.56 ***	10.90 ***	0.46 ^ns^	11.83 ***	0.29 ^ns^	0.67 ^ns^
HI	82.70 *	3.15 ***	2.53 **	12.50 ^ns^	2.99 ***	1.90 *	1.01 ^ns^	34.18 **	5.22 ***	0.93 ^ns^	3.67 ***	0.93 ^ns^	0.79 ^ns^

Na^+^, K^+^, and Ca^2+^: content of sodium, potassium, and calcium (mmol kg^−1^ DW), respectively; RWC: relative water content (%); CT: canopy temperature (°C); Fv/Fm: maximum quantum PSII photochemical efficiency; Chla/Chlb: chlorophyll a/b ratio; Chlt: total chlorophyll content (mg g^−1^ FW); PDW: plant dry weight (g plant^−1^); GLA: green leaf area (cm^2^ plant^−1^); GNPS: grain number per spike; BY: biological yield (ton m^2^); GY: grain yield (ton m^2^); and HI: harvest index (%). * *p* ≤ 0.05, ** *p* ≤ 0.01, *** *p* ≤ 0.001, ns—not significant.

**Table 2:** Mean values for ion content in the shoots of 22 wheat genotypes under control (C) and salinity stress (S) conditions across two years.

Varieties/RILs	Na^+^ (mmol kg^−1^ DW^−1^)		K^+^ (mmol kg^−1^ DW)		Ca^2+^ (mmol kg^−1^ DW^−1^)		K^+^/Na^+^		Ca^2+^/Na^+^	
	C	S	C	S	C	S	C	S	C	S
Sakha 93	103.0 ^a^	1737.2 ^c–f^	1163.7 ^a–d^	969.1 ^def^	448.1 ^bcd^	520.9 ^cde^	11.3 ^efg^	0.55 ^ab^	4.36 ^hij^	0.30 ^a^
Sids 1	87.8 ^a^	2026.5 ^abc^	1034.5 ^de^	904.8 ^ef^	461.3 ^bc^	476.7 ^ef^	11.8 ^de^	0.45 ^ab^	5.25 ^cde^	0.24 ^a^
Sakha 61	95.9 ^a^	2279.9 ^a^	1236.9 ^ab^	744.9 ^gh^	457.7 ^bc^	450.4 ^f^	12.9 ^bc^	0.32 ^b^	4.77 ^fgh^	0.20 ^a^
Kawz	88.4 ^a^	1404.6 ^f^	1058.4 ^d^	1076.3 ^d^	430.4 ^cd^	539.9 ^cd^	12.0 ^de^	0.75 ^ab^	4.88 ^e–h^	0.38 ^a^
RIL1-1	91.1 ^a^	2059.1 ^abc^	1002.8 ^de^	719.5 ^h^	422.5 ^d^	448.6 ^f^	11.0 ^fg^	0.35 ^b^	4.65 ^gh^	0.22 ^a^
RIL1-2	96.4 ^a^	1853.0 ^cd^	1198.9 ^ab^	845.9 ^fg^	476.4 ^b^	509.4 ^e^	12.4 ^cd^	0.47 ^ab^	4.95 ^efg^	0.28 ^a^
RIL1-3	141.5 ^a^	2199.4 ^a^	1245.6 ^a^	846.5 ^fg^	488.6 ^ab^	480.7 ^e^	8.8 ^j^	0.38 ^b^	3.46 ^k^	0.22 ^a^
RIL1-4	91.1 ^a^	2115.5 ^a^	1169.3 ^abc^	890.6 ^f^	481.9 ^ab^	490.0 ^e^	12.9 ^bc^	0.42 ^b^	5.30 ^cd^	0.23 ^a^
RIL1-5	106.4 ^a^	2104.3 ^a^	1155.3 ^bcd^	737.9 ^h^	455.2 ^bc^	446.0 ^f^	10.9 ^g^	0.35 ^b^	4.28 ^ij^	0.21 ^a^
RIL1-6	108.9 ^a^	1713.8 ^def^	1195.8 ^ab^	1221.9 ^bc^	458.2 ^bc^	557.1 ^c^	11.0 ^fg^	0.72 ^ab^	4.22 ^ij^	0.32 ^a^
RIL1-7	83.9 ^a^	1580.6 ^f^	1101.9 ^cd^	1109.3 ^d^	438.0 ^cd^	513.1 ^e^	13.2 ^b^	0.75 ^ab^	5.21 ^cde^	0.34 ^a^
RIL2-1	132.2 ^a^	2073.5 ^ab^	1019.6 ^de^	997.9 ^de^	448.3 ^bc^	493.6 ^e^	7.7 ^l^	0.48 ^ab^	3.38 ^k^	0.24 ^a^
RIL2-2	82.7 ^a^	1967.0 ^bc^	924.1 ^ef^	954.7 ^ef^	419.2 ^d^	487.2 ^e^	11.4 ^ef^	0.49 ^ab^	5.04 ^ef^	0.25 ^a^
RIL2-3	68.8 ^a^	1776.9 ^cde^	639.5 ^i^	1069.7 ^d^	405.6 ^de^	516.8 ^de^	9.3 ^i^	0.60 ^ab^	5.87 ^b^	0.29 ^a^
RIL2-4	90.8 ^a^	1608.4 ^f^	747.8 ^gh^	1218.8 ^bc^	400.6 ^e^	536.3 ^cd^	8.3 ^k^	0.76 ^ab^	4.41 ^hi^	0.33 ^a^
RIL2-5	105.5 ^a^	1702.3 ^ef^	725.6 ^gh^	1222.9 ^bc^	426.5 ^d^	532.5 ^cd^	6.9 ^n^	0.71 ^ab^	4.02 ^j^	0.31 ^a^
RIL2-6	68.7 ^a^	1480.4 ^f^	902.3 ^f^	1020.5 ^de^	434.8 ^cd^	507.8 ^e^	13.2 ^b^	0.72 ^ab^	6.33 ^a^	0.35 ^a^
RIL2-7	87.5 ^a^	1486.2 ^f^	1231.5 ^ab^	1113.5 ^d^	467.6 ^b^	552.7 ^c^	14.1 ^a^	0.76 ^ab^	5.35 ^c^	0.37 ^a^
RIL2-8	96.2 ^a^	1509.1 ^f^	1038.2 ^d^	1175.4 ^c^	497.4 ^a^	558.7 ^c^	10.8 ^g^	0.78 ^ab^	5.17 ^de^	0.37 ^a^
RIL2-9	105.3 ^a^	1932.2 ^c^	769.2 ^g^	1016.6 ^de^	419.1 ^d^	504.8 ^e^	7.3 ^m^	0.53 ^ab^	3.98 ^j^	0.26 ^a^
RIL2-10	105.5 ^a^	1599.4 ^f^	933.7 ^ef^	1287.3 ^a^	453.5 ^bc^	588.8 ^a^	8.9 ^j^	0.80 ^ab^	4.30 ^ij^	0.38 ^a^
RIL2-11	71.3 ^a^	1461.1 ^f^	708.2 ^h^	1244.8 ^ab^	423.6 ^d^	573.6 ^b^	9.9 ^h^	0.85 ^ab^	5.93 ^b^	0.39 ^a^
Average	95.85	1803.20	1009.2	1017.7	446.1	512.98	10.7	0.59	4.78	0.30

Mean values with the same letter are not significantly different at a significance level of 0.05, based on Tukey’s test.

**Table 3:** Mean values of physiological traits measured for 22 wheat genotypes under control (C) and salinity stress (S) conditions across two years.

Varieties/RILs	RWC		CT (°C)		F_v_/F_m_		Chla/Chlb		Chlt	
	C	S	C	S	C	S	C	S	C	S
Sakha 93	79.78 ^a^	69.00 ^cd^	27.67 ^f^	36.48 ^ae^	0.81 ^abc^	0.83 ^ab^	2.13 ^fgh^	2.79 ^abc^	4.18 ^cde^	3.26 ^abc^
Sids 1	76.28 ^abc^	66.58 ^d^	27.73 ^f^	36.51 ^a–e^	0.81 ^abc^	0.79 ^a–f^	2.27 ^fgh^	2.04 ^c–h^	3.92 ^def^	2.67 ^cde^
Sakha 61	79.17 ^a^	62.12 ^d^	28.01 ^f^	39.06 ^a^	0.80 ^a–d^	0.72 ^f^	4.73 ^a^	1.31 ^h^	3.59 ^f^	2.13 ^f^
Kawz	81.68 ^a^	65.52 ^d^	27.76 ^f^	35.79 ^a–e^	0.81 ^a–d^	0.78 ^a–f^	2.51 ^fg^	2.08 ^c–g^	4.87 ^ab^	3.34 ^ab^
RIL1-1	79.47 ^a^	62.57 ^d^	26.00 ^f^	38.74 ^a^	0.81 ^a–d^	0.74 ^def^	3.84 ^bc^	1.83 ^e–h^	3.57 ^f^	2.59 ^de^
RIL1-2	83.39 ^a^	63.98 ^d^	27.11 ^f^	37.36 ^abc^	0.83 ^a^	0.78 ^a–f^	3.31 ^de^	1.64 ^gh^	4.03 ^de^	2.71 ^cde^
RIL1-3	81.56 ^a^	65.18 ^d^	26.24 ^f^	38.16 ^ab^	0.82 ^abc^	0.78 ^a–f^	4.15 ^b^	1.78 ^fgh^	3.96 ^de^	2.71 ^cde^
RIL1-4	80.81 ^a^	64.83 ^d^	25.83 ^f^	38.08 ^ab^	0.81 ^a–d^	0.78 ^a–f^	3.49 ^cd^	1.52 ^h^	4.16 ^de^	2.54 ^e^
RIL1-5	81.14 ^a^	65.33 ^d^	26.38 ^f^	38.06 ^ab^	0.82 ^abc^	0.77 ^a–f^	2.97 ^ef^	1.92 ^d–h^	3.75 ^ef^	2.62 ^de^
RIL1-6	83.98 ^a^	69.68 ^bcd^	26.31 ^f^	33.79 ^de^	0.82 ^abc^	0.84 ^a–f^	1.98 ^h^	2.82 ^ab^	4.45 ^c^	3.68 ^a^
RIL1-7	83.84 ^a^	66.64 ^d^	25.76 ^f^	35.74 ^a–e^	0.81 ^a–d^	0.81 ^a–f^	2.35 ^fg^	2.25 ^cde^	4.89 ^a^	3.71 ^a^
RIL2-1	78.89 ^a^	65.06 ^d^	28.01 ^f^	37.44 ^abc^	0.82 ^abc^	0.75 ^a^	2.24 ^fgh^	2.27 ^cd^	3.83 ^ef^	2.76 ^cde^
RIL2-2	79.65 ^a^	65.92 ^d^	27.91 ^f^	38.24 ^ab^	0.80 ^a–e^	0.73 ^a–d^	2.72 ^f^	2.22 ^cde^	3.85 ^ef^	2.94 ^c^
RIL2-3	78.95 ^a^	66.17 ^d^	26.76 ^f^	36.96 ^a–d^	0.79 ^a–f^	0.78 ^c–f^	2.38 ^fg^	2.30 ^c^	4.18 ^de^	2.84 ^cd^
RIL2-4	81.80 ^a^	67.23 ^d^	28.37 ^f^	35.14 ^b–e^	0.79 ^a–f^	0.79 ^ef^	1.67 ^h^	2.63 ^bc^	4.11 ^de^	3.09 ^bc^
RIL2-5	80.41 ^a^	67.84 ^d^	27.59 ^f^	35.04 ^b–e^	0.82 ^abc^	0.77 ^a–f^	1.72 ^h^	2.66 ^bc^	4.42 ^c^	3.22 ^bc^
RIL2-6	81.29 ^a^	65.17 ^d^	26.21 ^f^	36.18 ^a–e^	0.79 ^a–f^	0.79 ^a–f^	2.39 ^fg^	2.11 ^c–f^	4.66 ^bc^	2.88 ^c^
RIL2-7	77.48 ^ab^	66.64 ^d^	27.98 ^f^	35.89 ^a–e^	0.82 ^abc^	0.77 ^a–f^	1.93 ^h^	2.98 ^a^	4.17 ^de^	3.33 ^ab^
RIL2-8	78.26 ^a^	69.69 ^bcd^	27.44 ^f^	34.58 ^cde^	0.79 ^a–f^	0.77 ^a–f^	1.77 ^h^	2.84 ^ab^	4.20 ^cd^	3.29 ^ab^
RIL2-9	80.73 ^a^	64.15 ^d^	27.24 ^f^	37.59 ^abc^	0.83 ^ab^	0.75 ^b–f^	2.47 ^fg^	2.24 ^cde^	4.14 ^de^	2.96 ^c^
RIL2-10	81.30 ^a^	69.33 ^cd^	26.41 ^f^	33.39 ^e^	0.80 ^a–e^	0.79 ^a–f^	2.58 ^f^	2.32 ^c^	4.86 ^ab^	3.73 ^a^
RIL2-11	82.00 ^a^	67.78 ^d^	26.88 ^f^	34.34 ^cde^	0.83 ^ab^	0.80 ^a–e^	2.11 ^gh^	2.38 ^c^	4.57 ^c^	3.43 ^a^
Average	80.54	66.20	27.07	36.48	0.81	0.78	2.62	2.22	4.20	3.02

Mean values with the same letter are not significantly different at a significance level of 0.05, based on Tukey’s test. RWC, CT, Fv/Fm, Chla/Chlb, and Chlt indicate relative water content (%), canopy temperature (°C), maximum quantum PSII photochemical efficiency, chlorophyll a/b ratio, and total chlorophyll content (mg g^−1^ FW), respectively.

**Table 4:** Mean values of different growth and yield traits measured for 22 wheat genotypes under control (C) and salinity stress (S) conditions across two years.

Varieties/RILs	PDW		GLA		GNPS		GY		BY		HI	
	C	S	C	S	C	S	C	S	C	S	C	S
Sakha93	5.44 ^e–k^	4.43 ^j–r^	192.5 ^f–k^	132.7 ^j–n^	48.95 ^b–f^	40.40 ^h–m^	0.54 ^d–h^	0.39 ^o–s^	1.8 ^b–i^	1.2 ^o–s^	30.5 ^a–k^	33.1 ^a–j^
Sids 1	5.58 ^d–i^	4.46 ^j–r^	185.93 ^f–l^	132.13 ^j–n^	49.11 ^a–f^	38.28 ^i–n^	0.54 ^e–i^	0.37 ^o–s^	1.7 ^c–l^	1.5 ^i–p^	32.7 ^a–j^	25.8 ^ijk^
Sakha61	5.74 ^c–g^	3.36 ^r^	218.62 ^d–h^	106.47 ^n^	49.48 ^a–e^	33.34 ^n^	0.58 ^b–g^	0.30 ^s^	1.6 ^f–n^	1 ^qrs^	37.4 ^a^	29.6 ^a–k^
RIL1-1	6.92 ^ab^	4.53 ^h–q^	305.41 ^ab^	156.37 ^h–n^	55.78 ^a^	40.90 ^h–l^	0.68 ^a^	0.41 ^m–r^	2.0 ^abc^	1.5 ^i–p^	34 ^a–g^	27.6 ^d–k^
RIL1-2	6.62 ^a–d^	3.51 ^pqr^	235.99 ^c–f^	119.71 ^mn^	51.56 ^abc^	34.86 ^lmn^	0.60 ^a–f^	0.33 ^rs^	1.9 ^a–d^	0.9 ^s^	31 ^a–k^	35.7 ^abc^
RIL1-3	6.76 ^abc^	4.38 ^k–r^	323.69 ^a^	130.99 ^j–n^	53.06 ^ab^	40.10 ^h–m^	0.67 ^ab^	0.36 ^o–s^	2.1 ^a^	1.3 ^l–r^	31.5 ^a–k^	28 ^c–k^
RIL1-4	6.99 ^a^	4.11 ^n–r^	272.97 ^a–d^	129.67 ^k–n^	52.74 ^ab^	37.18 ^j–n^	0.63 ^a–d^	0.34 ^qrs^	2.2 ^a^	1.5 ^i–p^	29.4 ^b–k^	24.1 ^k^
RIL1-5	6.44 ^a–e^	3.64 ^o–r^	313.39 ^ab^	125.50 ^lmn^	52.23 ^abc^	36.79 ^k–n^	0.65 ^abc^	0.35 ^p–s^	2.03 ^ab^	1.0 ^rs^	31.9 ^a–k^	37.0 ^ab^
RIL1-6	5.23 ^f–m^	3.51 ^qr^	198.44 ^e–i^	126.72 ^lmn^	49.23 ^a–f^	38.44 ^i–n^	0.55 ^d–h^	0.36 ^p–s^	1.9 ^a–g^	1.4 ^j–q^	29.5 ^a–k^	26.4 ^f–k^
RIL1-7	5.37 ^e–k^	4.75 ^f–n^	290.64 ^ab^	150.54 ^i–n^	53.57 ^ab^	44.36 ^d–i^	0.66 ^a–e^	0.44 ^i–p^	1.9 ^a–e^	1.5 ^i–p^	32.5 ^a–j^	29.5 ^a–k^
RIL2-1	6.90 ^ab^	4.75 ^f–n^	304.81 ^ab^	145.32 ^i–n^	53.78 ^ab^	42.36 ^f–k^	0.68 ^a^	0.42 ^k–r^	2.0 ^ab^	1.6 ^e–n^	33.6 ^a–i^	26.7 ^f–k^
RIL2-2	5.25 ^f–l^	4.18 ^l–r^	172.00 ^g–m^	144.41 ^i–n^	45.90 ^c–h^	33.95 ^mn^	0.51 ^g–l^	0.36 ^p–s^	1.5 ^h–p^	1.3 ^k–q^	34.0 ^a–g^	27.2 ^e–k^
RIL2-3	5.23 ^f–m^	4.13 ^m–r^	160.44 ^h–n^	137.63 ^i–n^	50.67 ^a–d^	36.17 ^k–n^	0.53 ^f–j^	0.36 ^p–s^	1.5 ^g–o^	1.2 ^p–s^	34.3 ^a–f^	31.1 ^a–k^
RIL2-4	5.15 ^f–n^	4.44 ^j–r^	142.74 ^i–n^	134.32 ^i–n^	48.57 ^b–g^	38.86 ^i–n^	0.50 ^g–m^	0.39 ^o–s^	1.5 ^g–o^	1.5 ^i–p^	32.9 ^a–j^	27.1 ^e–k^
RIL2-5	5.03 ^f–n^	4.61 ^h–p^	161.68 ^g–n^	142.80 ^i–n^	47.96 ^b–g^	39.58 ^h–n^	0.50 ^g–n^	0.41 ^l–r^	1.6 ^d–n^	1.6 ^d–m^	30.9 ^a–k^	25.5 ^jk^
RIL2-6	5.85 ^b–f^	4.54 ^h–q^	252.19 ^b–e^	157.87 ^h–n^	49.04 ^a–f^	38.52 ^i–n^	0.53 ^e–i^	0.41 ^m–r^	1.7 ^b–j^	1.6 ^e–n^	31.8 ^a–k^	26 ^h–k^
RIL2-7	5.62 ^d–h^	4.64 ^g–o^	194.10 ^f–j^	149.01 ^i–n^	53.26 ^ab^	40.83 ^h–l^	0.58 ^b–g^	0.39 ^o–s^	1.9 ^a–f^	1.5 ^g–p^	31 ^a–k^	25.6 ^jk^
RIL2-8	5.73 ^c–g^	4.69 ^g–o^	253.22 ^b–e^	144.94 ^i–n^	48.09 ^b–g^	40.36 ^h–m^	0.52 ^f–j^	0.40 ^n–r^	1.5 ^g–p^	1.4 ^j–p^	34.9 ^a–e^	28.5 ^c–k^
RIL2-9	5.02 ^f–n^	4.70 ^g–o^	186.93 ^f–l^	151.74 ^i–n^	49.69 ^a–e^	41.79 ^g–k^	0.52 ^f–k^	0.41 ^m–r^	1.5 ^f–o^	1.4 ^j–q^	33.8 ^a–h^	29.8 ^a–k^
RIL2-10	6.44 ^a–e^	4.06 ^n–r^	223.33 ^d–g^	146.73 ^i–n^	48.80 ^b–f^	36.31 ^k–n^	0.54 ^d–h^	0.37 ^o–s^	1.6 ^d–n^	1.4 ^j–p^	34.1 ^a–g^	26.3 ^g–k^
RIL2-11	6.73 ^abc^	5.40 ^e–k^	281.23 ^a–d^	156.24 ^h–n^	52.65 ^ab^	43.67 ^e–j^	0.65 ^abc^	0.46 ^h–o^	1.9 ^a–h^	1.3 ^n–s^	35.3 ^a–d^	36.9 ^ab^
Average	5.89	4.33	232.36	139.55	50.68	38.99	0.58	0.38	1.78	1.35	32.79	29.09

Mean values with the same letter are not significantly different at a significance level of 0.05, based on Tukey’s test. PDW, GLA, GNPS, GY, BY, and HI indicate plant dry weight (g plant^−1^), green leaf area (cm^2^ plant^−1^), grain number per spike, biological yield (ton m^2^), grain yield (ton m^2^), and harvest index (%), respectively.

**Table 5:** Eigenvalue, variability, cumulative variability, and factor loadings of the first five and two principal components for the different traits of 22 wheat genotypes grown under the control and salinity stress conditions, respectively.

Traits	Control					Salinity Stress	
	PC1	PC2	PC3	PC4	PC5	PC1	PC2
Na^+^	0.18	**0.90**	−0.25	0.12	−0.12	**−0.89**	0.00
K^+^	0.54	0.46	**0.60**	0.07	−0.26	**0.93**	0.01
Ca^2+^	0.42	0.43	**0.52**	0.10	−0.43	**0.93**	0.20
K^+^/Na^+^	0.37	−0.35	**0.79**	−0.02	−0.16	**0.95**	0.01
Ca^2+^/Na^+^	−0.05	**−0.84**	0.43	−0.15	−0.02	**0.92**	0.09
RWC	**0.66**	−0.19	−0.50	−0.04	−0.07	**0.85**	0.14
CT	**−0.67**	0.18	0.11	0.45	−0.02	**−0.96**	−0.08
Fv/Fm	0.31	0.45	−0.19	0.23	−0.04	**0.66**	0.00
Chl a/b	0.45	0.37	0.41	−0.23	**0.63**	**0.79**	−0.07
Chlt	0.35	**−0.64**	−0.39	0.36	−0.31	**0.93**	0.12
DW	**0.81**	0.05	−0.11	0.18	0.36	**0.92**	−0.06
GLA	**0.90**	−0.01	−0.05	0.33	−0.04	**0.87**	−0.16
GNPS	**0.85**	−0.38	−0.02	0.00	0.03	**0.88**	0.07
GY	**0.97**	−0.13	−0.01	0.12	0.11	**0.97**	0.09
BY	**0.93**	0.04	−0.12	−0.28	−0.06	0.61	**−0.78**
HI	−0.21	−0.22	0.30	0.79	0.33	−0.02	**0.99**
Eigenvalue	5.97	3.07	2.25	1.36	1.05	11.56	1.73
Variability (%)	37.32	19.16	14.07	8.49	6.56	72.25	10.78
Cumulative %	37.32	56.48	70.55	79.03	85.59	72.25	83.03

Values in bold denote traits for the suggested factor name. Na^+^, K^+^, and Ca^2+^: contents of sodium, potassium, and calcium (mmol kg^−1^ DW), respectively; RWC: relative water content (%); CT: canopy temperature (°C); Fv/Fm: maximum quantum PSII photochemical efficiency; Chla/Chlb: chlorophyll a/b ratio; Chlt: total chlorophyll content (mg g^−1^ FW); PDW: plant dry weight (g plant^−1^); GLA: green leaf area (cm^2^ plant^−1^); GNPS: grain number per spike; BY: biological yield (ton m^2^); GY: grain yield (ton m^2^); and HI: harvest index (%).

**Table 6:** Comparison profile of the three groups of genotypes identified by two-way clustering heatmaps, based on all traits under both control and salinity stress conditions.

	Control			Salinity		
Group	Group 1	Group 2	Group 3	Group 1	Group 2	Group 3
Gen. No.	7	6	9	11	8	3
Na^+^	102.24	97.55	89.76	1571.18	1991.63	2151.46
K^+^	1129.09	804.30	1052.59	1150.89	921.75	785.01
Ca^2+^	461.02	419.87	452.02	543.77	489.40	462.97
K^+^/Na^+^	11.30	8.48	11.78	0.74	0.47	0.36
Ca^2+^/Na^+^	4.62	4.45	5.12	0.35	0.25	0.22
RWC	82.37	80.07	79.43	67.68	65.30	63.17
CT	26.49	27.65	27.14	35.12	37.54	38.63
Fv/Fm	0.81	0.81	0.81	0.79	0.77	0.74
Chla/Chlb	2.91	2.20	2.68	2.53	2.05	1.55
Chlt	4.46	4.09	4.07	3.36	2.78	2.42
PDW	6.59	5.49	5.61	4.69	4.16	3.51
GLA	298.88	185.40	211.94	148.71	135.33	117.23
GNPS	53.40	48.49	50.03	41.23	37.41	35.00
GY	654.53	518.49	556.23	414.47	362.30	330.18
BY	2021.48	1577.80	1715.70	1438.09	1367.26	979.76
HI	32.62	33.01	32.78	29.25	26.98	34.12

Na^+^, K^+^, and Ca^2+^: contents of sodium, potassium, and calcium (mmol kg^−1^ DW), respectively; RWC: relative water content (%); CT: canopy temperature (°C); Fv/Fm: maximum quantum PSII photochemical efficiency; Chla/Chlb: chlorophyll a/b ratio; Chlt: total chlorophyll content (mg g^−1^ FW); PDW: plant dry weight (g plant^−1^); GLA: green leaf area (cm^2^ plant^−1^); GNPS: grain number per spike; BY: biological yield (ton m^2^); GY: grain yield (ton m^2^); and HI: harvest index (%).

## Data Availability

All data are presented within the article.

## References

[1] Kotula L., Zahra N., Farooq M., Shabala S., Siddique K.H. (2024). Making wheat salt tolerant: What is missing?. Crop J..

[2] Chaurasia S., Kumar A., Singh A.K. (2022). Comprehensive evaluation of morpho-physiological and ionic traits in wheat (*Triticum aestivum* L.) genotypes under salinity stress. Agriculture.

[3] Shokat S., Großkinsky D.K. (2019). Tackling salinity in sustainable agriculture-what developing countries may learn from approaches of the developed world. Sustainability.

[4] Shiferaw B., Smale M., Braun H.J., Duveiller E., Reynolds M., Muricho G. (2013). Crops that feed the world 10. Past successes and future challenges to the role played by wheat in global food security. Food Secur..

[5] Dadshani S., Sharma R.C., Baum M., Ogbonnaya F.C., Léon J., Ballvora A. (2019). Multi-dimensional evaluation of response to salt stress in wheat. PLoS ONE.

[6] El-Hendawy S., Al-Suhaibani N., Mubushar M., Tahir M.U., Marey S., Refay Y., Tola E. (2022). Combining hyperspectral reflectance and multivariate regression models to estimate plant biomass of advanced spring wheat lines in diverse phenological stages under salinity conditions. Appl. Sci..

[7] Marone D., Russo M.A., Mores A., Ficco D.B.M., Laidò G., Mastrangelo A.M., Borrelli G.M. (2021). Importance of landraces in cereal breeding for stress tolerance. Plants.

[8] Oyiga B.C., Sharma R.C., Shen J., Baum M., Ogbonnaya F.C., Leon J., Ballvora A. (2016). Identification and characterization of salt tolerance of wheat germplasm using a multivariable screening approach. J. Agron. Crop Sci..

[9] Quan X., Liang X., Li H., Xie C., He W., Qin Y. (2021). Identification and characterization of wheat germplasm for salt tolerance. Plants.

[10] Tavakkoli E., Rengasamy P., McDonald G.K. (2010). High concentrations of Na^+^ and Cl-ions in soil solution have simultaneous detrimental effects on growth of faba bean under salinity stress. J. Exp. Bot..

[11] El-Hendawy S.E., Hassan W.M., Al-Suhaibani N.A., Refay Y., Abdella K.A. (2017). Comparative performance of multivariable agro-physiological parameters for detecting salt tolerance of wheat cultivars under simulated saline field growing conditions. Front. Plant Sci..

[12] Füzy A., Kovács R., Cseresnyés I., Parádi I., Szili-Kovács T., Kelemen B., Rajkai K., Takács T. (2019). Selection of plant physiological parameters to detect stress effects in pot experiments using principal component analysis. Acta Physiol. Plant..

[13] Al-Ashkar I., Alderfasi A., El-Hendawy S.E., Al-Suhaibani N., El-Kafafi S., Seleiman M.F. (2019). Detecting salt tolerance in doubled haploid wheat Lines. Agronomy.

[14] Mubushar M., El-Hendawy S., Tahir M.U., Alotaibi M., Mohammed N., Refay Y., Tola E. (2022). Assessing the suitability of multivariate analysis for stress tolerance indices, biomass, and grain yield for detecting salt tolerance in advanced spring wheat lines irrigated with saline water under field conditions. Agronomy.

[15] Singh M., Kumar J., Singh S., Singh V.P., Prasad S.M. (2015). Roles of osmoprotectants in improving salinity and drought tolerance in plants: A review. Rev. Environ. Sci. Bio/Technol..

[16] Pongprayoon W., Tisarum R., Theerawittaya C., Cha-Um S. (2019). Evaluation and clustering on salt-tolerant ability in rice genotypes (*Oryza sativa* L. subsp. indica) using multivariate physiological indices. Physiol. Mol. Biol. Plants.

[17] Tao R., Ding J., Li C., Zhu X., Guo W., Zhu M. (2021). Evaluating and screening of agro-physiological indices for salinity stress tolerance in wheat at the seedling stage. Front. Plant Sci..

[18] Matković Stojšin M., Petrović S., Banjac B., Zečević V., Roljević Nikolić S., Majstorović H., Đorđević R., Knežević D. (2022). Assessment of genotype stress tolerance as an effective way to sustain wheat production under salinity stress conditions. Sustainability.

[19] Kaya Y. (2022). Phenotyping winter wheat for early ground cover. Czech J. Genet. Plant Breed..

[20] Zhang L., Liu G., Zhao G. (2014). Characterization of a wheat R2R3-MYB transcription factor gene, TaMYB19, involved in enhanced abiotic stresses in arabidopsis. Plant Cell Physiol..

[21] Tester M., Davenport R. (2003). Na^+^ tolerance and Na^+^ transport in higher plants. Ann. Bot..

[22] Khan M.K., Pandey A., Hamurcu M., Vyhnánek T., Zargar S.M., Kahraman A., Topal A., Gezgin S. (2024). Exploring strigolactones for inducing abiotic stress tolerance in plants. Czech J. Genet. Plant Breed..

[23] Munns R., James R.A., Läuchli A. (2006). Approaches to increasing the salt tolerance of wheat and other cereals. J. Exp. Bot..

[24] Wu D.Z., Sato K., Ma J.F. (2015). Genome-wide association mapping of cadmium accumulation in different organs of barley. New Phytol..

[25] Ouhaddach M., ElYacoubi H., Douaik A., Rochdi A. (2018). Morpho-physiological and biochemical responses to salt stress in wheat (*Triticum aestivum* L.) at the heading stage. J. Mater. Environ. Sci..

[26] El-Hendawy S., Al-Suhaibani N., Dewir Y.H., Elsayed S., Alotaibi M., Hassan W., Refay Y., Tahir M.U. (2019). Ability of modified spectral reflectance indices for estimating growth and photosynthetic efficiency of wheat under saline field conditions. Agronomy.

[27] Zarco-Tejada P.J., Pushnik J.C., Dobrowski S., Ustin S.L. (2003). Steady-state chlorophyll a fluorescence detection from canopy derivative reflectance and double-peak red-edge effects. Remote Sens. Environ..

[28] Munns R., Tester M. (2008). Mechanisms of salinity tolerance. Annu. Rev. Plant Biol..

[29] Orzechowska A., Trtílek M., Tokarz K.M., Szymańska R., Niewiadomska E., Rozpądek P., Wątor K. (2021). Thermal analysis of stomatal response under salinity and high light. Int. J. Mol. Sci..

[30] Sirault X.R.R., James R.A., Furbank R.T., Sirault X.R.R., James R.A., Furbank R.T. (2009). A New screening method for osmotic component of salinity tolerance in cereals using infrared thermography. Funct. Plant Biol..

[31] Radi A.A., Farghaly F.A., Hamada A.M. (2013). Physiological and biochemical responses of salt-tolerant and salt-sensitive wheat and bean genotypes to salinity. J. Biol. Earth Sci..

[32] Genc Y., Taylor J., Lyons G., Li Y., Cheong J., Appelbee A., Oldach K., Sutton T. (2019). Bread wheat with high salinity and sodicity tolerance. Front. Plant Sci..

[33] Khan M., Rahman M., Hasan M., Amin M.F., Matin M.Q.I., Faruq G., Alkeridis L.A., Gaber A., Hossain A. (2024). Assessment of the salt tolerance of diverse bread wheat (*Triticum aestivum* L.) genotypes during the early growth stage under hydroponic culture conditions. Heliyon.

[34] Acosta-Motos J.R., Ortuño M.F., Bernal-Vicente A., Diaz-Vivancos P., Sanchez-Blanco M.J., Hernandez J.A. (2017). Plant responses to salt stress: Adaptive mechanisms. Agronomy.

[35] Thiel V., Hügler M., Blümel M., Baumann H.I., Gärtner A., Schmaljohann R., Strauss H., Garbe-Schönberg D., Petersen S., Cowart D.A. (2012). Widespread occurrence of two carbon fixation pathways in tubeworm endosymbionts: Lessons from hydrothermal vent associated tubeworms from the Mediterranean Sea. Front. Microbiol..

[36] Basu S., Giri R.K., Benazir I., Kumar S., Rajwanshi R., Dwivedi S.K., Kumar G. (2017). Comprehensive physiological analyses and reactive oxygen species profiling in drought tolerant rice genotypes under salinity stress. Physiol. Mol. Biol. Plants.

[37] Basu S., Kumar A., Benazir I., Kumar G. (2021). Reassessing the role of ion homeostasis for improving salinity tolerance in crop plants. Physiol. Plant.

[38] Du Y.D., Zhang Q., Cui B.J., Sun J., Wang Z., Ma L.H., Niu W.Q. (2020). Aerated irrigation improves tomato yield and nitrogen use efficiency while reducing nitrogen application rate. Agric. Water Manag..

[39] Liu X., Chang X., Wang Y., Wang D., Wang X., Meng Q., Wang P. (2024). Adaptation to priming drought at six-leaf stage relieves maize yield loss to individual and combined drought and heat stressors around flowering. Environ. Exp. Bot..

[40] Grzesiak S., Hordy´nska N., Szczyrek P., Grzesiak M.T., Noga A., Szechy’nska-Hebda M. (2019). Variation among wheat (*Triticum easativum* L.) genotypes in response to the drought stress: I-Selection approaches. J. Plant Interact..

[41] Mohi-Ud-Din M., Hossain M.A., Rohman M.M., Uddin M.N., Haque M.S., Ahmed J.U., Hossain A., Hassan M.M., Mostofa M.G. (2021). Multivariate analysis of morpho-physiological traits reveals differential drought tolerance potential of bread wheat genotypes at the seedling stage. Plants.

[42] El-Hendawy S.E., Hu Y., Yakout G.M., Awad A.M., Hafiz S.E., Schmidhalter U. (2005). Evaluating salt tolerance of wheat genotypes using multiple parameters. Eur. J. Agron..

[43] El-Hendawy S., Ruan Y., Hu Y., Schmidhalter U. (2009). A comparison of screening criteria for salt tolerance in wheat under field and controlled environmental conditions. J. Agron. Crop Sci..

[44] Zadoks J.C., Chang T.T., Konzak C.F. (1974). A decimal code for the growth stages of cereals. Weed Res..

[45] Motsara M.R., Roy R.N. (2008). Guide to Laboratory Establishment for Plant Nutrient Analysis.

[46] Lichtenthaler H.K., Wellburn A.R. (1983). Determinations of total carotenoids and chlorophylls A and B of leaf extracts in different solvents. Biochem. Soc. Trans..

[47] Saddiq M.S., Iqbal S., Hafeez M.B., Ibrahim A.M., Raza A., Fatima E.M., Baloch H., Woodrow P., Ciarmiello L.F. (2021). Effect of salinity stress on physiological changes in winter and spring wheat. Agronomy.

[48] Ashraf M.A., Hafeez A., Rasheed R., Hussain I., Farooq U., Rizwan M., Ali S. (2023). Evaluation of physio-morphological and biochemical responses for salt tolerance in wheat (*Triticum aestivum* L.) cultivars. J. Plant Growth Regul..

[49] Toka H., Mouad B., Kebaili F.F., Maroua H., Awatef G., Hamdi B. (2024). Assessment of salt tolerance in Algerian oasis wheat landraces: An examination of biochemical, physiological, and agronomical traits. Emir. J. Food Agric..

[50] Xu Y., Bu W., Xu Y., Fei H., Zhu Y., Ahmad I., Nimir N.E.A., Zhou G., Zhu G. (2024). Effects of salt stress on physiological and agronomic traits of rice genotypes with contrasting salt tolerance. Plants.

[51] Anschütz U., Becker D., Shabala S. (2014). Going beyond nutrition: Regulation of potassium homoeostasis as a common denominator of plant adaptive responses to environment. J. Plant Physiol..

[52] Cherel I., Lefoulon C., Boeglin M., Sentenac H. (2014). Molecular mechanisms involved in plant adaptation to low K^+^ availability. J. Exp. Bot..

[53] Zhu M., Shabala S., Shabala L., Fan Y., Zhou M.X. (2016). Evaluating predictive values of various physiological indices for salinity stress tolerance in wheat. J. Agron. Crop Sci..

[54] Nedjimi B., Daoud Y. (2009). Ameliorative effect of CaCl_2_ on growth, membrane permeability and nutrient uptake in *Atriplex halimus* subsp. schweinfurthii grown at high (NaCl) salinity. Desalination.

[55] Shahid M.A., Sarkhosh A., Khan N., Balal R.M., Ali S., Rossi L., Gómez C., Mattson N., Nasim W., Garcia-Sanchez F. (2020). Insights into the physiological and biochemical impacts of salt stress on plant growth and development. Agronomy.

[56] Pour-Aboughadareh A., Mehrvar M.R., Sanjani S., Amini A., Nikkhah-Chamanabad H., Asadi A. (2021). Effects of salinity stress on seedling biomass, physiochemical properties, and grain yield in different breeding wheat genotypes. Acta Physiol. Plant..

[57] Boopal J., Sathee L., Ramasamy R., Pandey R., Chinnusamy V. (2023). Influence of incremental short-term salt stress at the seedling stage on root plasticity, shoot thermal profile and ion homeostasis in contrasting wheat genotypes. Agriculture.

[58] Ibrahimova U., Kumari P., Yadav S., Rastogi A., Antala M., Suleymanova Z., Zivcak M., Arif T.-U., Hussain S., Abdelhamid M. (2021). Progress in understanding salt stress response in plants using biotechnological tools. J. Biotechnol..

[59] Baker N.R., Rosenqvist E. (2004). Applications of chlorophyll fluorescence can improve crop production strategies: An examination of future possibilities. J. Exp. Bot..

[60] Chen B., Bian X., Tu M., Yu T., Jiang L., Lu Y., Chen X. (2023). Moderate salinity stress increases the seedling biomass in oilseed rape (*Brassica napus* L.). Plants.

[61] Amirjani M.R. (2011). Effect of salinity stress on growth; sugar content; pigments and enzyme activity of rice. Int. J. Bot. Stud..

[62] Wang X., Hou L., Lu Y., Wu B., Gong X., Liu M., Wang J., Sun Q., Vierling E., Xu S. (2018). Metabolic adaptation of wheat grain contributes to a stable filling rate under heat stress. J. Exp. Bot..

[63] Bayoumi T.Y., El-Hendawy S., Yousef M.S., Emam M.A., Okasha S.A. (2014). Application of infrared thermal imagery for monitoring salt tolerant of wheat genotypes. J. Am. Sci..

[64] Hairmansis A., Berger B., Tester M., Roy S.J. (2014). Image-based phenotyping for non-destructive screening of different salinity tolerance traits in rice. Rice.

[65] Jamil M., Bashir S., Anwar S., Bibi S., Bangash A., Ullah F., Shikrha E. (2012). Effect of salinity on physiological and biochemical characteristics of different varieties of rice. Pak. J. Bot..

[66] Azizpour K., Shakiba M.R., Sima N.A.K.K., Alyari H., Mogaddam M., Esfandiari E., Pessarakli M. (2010). Physiological response of spring durum wheat genotypes to salinity. J. Plant Nutr..

[67] Maghsoudi K., Emam Y., Ashraf M. (2015). Influence of foliar application of silicon on chlorophyll fluorescence, photosynthetic pigments, and growth in water-stressed wheat cultivars differing in drought tolerance. Turk. J. Bot..

[68] Ahmad Z., Waraich E.A., Akhtar S., Anjum S., Ahmad T., Mahboob W., Hafeez O.B.A., Tapera T., Labuschagne M., Rizwan M. (2018). Physiological responses of wheat to drought stress and its mitigation approaches. Acta Physiol. Plant..

[69] Irshad A., Ahmed R.I., Ur Rehman S., Sun G., Ahmad F., Sher M.A., Aslam M.Z., Hassan M.M., Qari S.H., Aziz M.K. (2022). Characterization of salt tolerant wheat genotypes by using morpho-physiological, biochemical, and molecular analysis. Front. Plant Sci..

[70] Aslam M., Ahmad K., Akhtar M.A., Maqbool M.A. (2017). Salinity Stress in Crop Plants: Effects of stress, tolerance mechanisms and breeding strategies for improvement. J. Agric. Basic Sci..

[71] Alam M.M., Khan M.A.R., Salehin Z.U., Uddin M., Soheli S.J., Khan T.Z. (2020). Combined PCA-Daugman method: An efficient technique for face and iris recognition. J. Adv. Math. Comput. Sci..

[72] Pastuszak J., Dziurka M., Hornyák M., Szczerba A., Kopec P., Płazek A. (2022). Physiological and biochemical parameters of salinity resistance of three durum wheat genotypes. Int. J. Mol. Sci..

